# Key predictors of mortality in profound hyponatremia beyond the correction rate

**DOI:** 10.1093/ckj/sfag219

**Published:** 2026-06-30

**Authors:** Koya Nagase, Takahiro Imaizumi, Atsushi Yamamori, Fumika N Nagase, Toshikazu Ozeki, Nobuhiro Nishibori, Takaya Ozeki, Hideaki Shimizu, Yoshiro Fujita, Kazuhiro Furuhashi, Tsuyoshi Watanabe, Shoichi Maruyama

**Affiliations:** Department of Nephrology, Nagoya University Graduate School of Medicine, Tsurumai-cho, Showa-ku, Nagoya, Aichi, Japan; Department of Nephrology, Nagoya University Graduate School of Medicine, Tsurumai-cho, Showa-ku, Nagoya, Aichi, Japan; Department of Clinical Research Education, Nagoya University Graduate School of Medicine, Tsurumai-cho, Showa-ku, Nagoya, Aichi, Japan; Department of Nephrology, Chubu Rosai Hospital, Komei-cho, Minato-ku, Nagoya, Aichi, Japan; Department of Rheumatology, Chubu Rosai Hospital, Komei-cho, Minato-ku, Nagoya, Aichi, Japan; Department of Nephrology, Chubu Rosai Hospital, Komei-cho, Minato-ku, Nagoya, Aichi, Japan; Department of Nephrology, Nagoya University Graduate School of Medicine, Tsurumai-cho, Showa-ku, Nagoya, Aichi, Japan; Department of Nephrology, Nagoya University Graduate School of Medicine, Tsurumai-cho, Showa-ku, Nagoya, Aichi, Japan; Department of Nephrology and Renal Replacement, Daido Hospital, Hakusui-cho, Minami-ku, Nagoya, Aichi, Japan; Department of Nephrology, Chubu Rosai Hospital, Komei-cho, Minato-ku, Nagoya, Aichi, Japan; Department of Rheumatology, Chubu Rosai Hospital, Komei-cho, Minato-ku, Nagoya, Aichi, Japan; Department of Nephrology, Nagoya University Graduate School of Medicine, Tsurumai-cho, Showa-ku, Nagoya, Aichi, Japan; Department of Rheumatology, Chubu Rosai Hospital, Komei-cho, Minato-ku, Nagoya, Aichi, Japan; Department of Internal Medicine, Kou Hospital, Hazenji, Kubo-cho, Toyokawa, Aichi, Japan; Department of Nephrology, Nagoya University Graduate School of Medicine, Tsurumai-cho, Showa-ku, Nagoya, Aichi, Japan

**Keywords:** correction rate, hyponatremia, machine learning, mortality, random forest

## Abstract

**Background:**

Current guidelines for hyponatremia recommend slow sodium correction; however, recent large-scale observational studies have associated slow correction with increased mortality. The association between correction rate and mortality can be influenced by numerous factors, including comorbidities, overall condition, and treatment interventions, but this complex interplay remains unclear. We aimed to clarify this association by developing interpretable machine-learning models using detailed clinical features.

**Methods:**

We included 546 patients with serum sodium ≤120 mEq/l, collected clinical features through chart review, and developed four machine-learning models to predict in-hospital mortality. The best-performing model, selected by the area under the receiver operating characteristic curve (ROC-AUC), was interpreted using SHapley Additive exPlanations (SHAP) to quantify each feature’s contribution to mortality prediction.

**Results:**

In-hospital mortality was 18%. The random forest model demonstrated the best predictive performance (ROC-AUC = 0.907; 95% confidence interval, 0.832–0.965). SHAP analysis revealed that the most influential predictors were baseline characteristics reflecting underlying illness severity: serum albumin, C-reactive protein, Charlson comorbidity index, and metastatic malignancy; their predictive effects were consistent across correction rates. Among treatment-related features, intravenous fluid choice and sodium monitoring frequency had a greater predictive impact than correction rate. Although slower correction was associated with higher mortality, the correction rate ranked 14th among all 59 features in predictive importance.

**Conclusions:**

In profound hyponatremia, multiple key predictors of mortality exist beyond the correction rate. These findings suggest that the observed association between slow correction and higher mortality may not be causal but rather an epiphenomenon driven by underlying illness severity and treatment intensity.

## Graphical Abstract

**Graphical Abstract:**
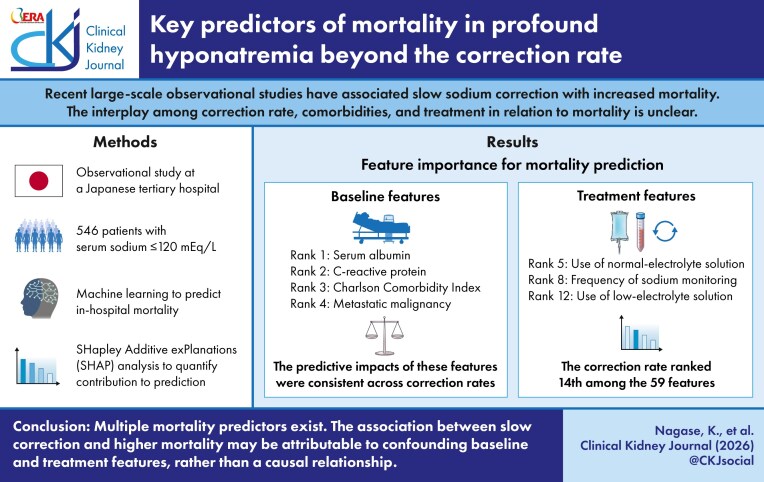

KEY LEARNING POINTS **What was known:**

- Current guidelines for hyponatremia recommend slow sodium correction; however, recent large-scale observational studies have associated slower correction with increased mortality, creating a clinical dilemma.
- The complex interplay among correction rate, comorbidities, overall condition, and treatment factors in relation to mortality remained unclear.

**This study adds:**

- Using machine learning, we demonstrated that the most influential predictors were baseline characteristics reflecting underlying illness severity; their predictive effects were consistent across correction rates.
- Among treatment-related features, intravenous fluid choice and sodium monitoring frequency outranked correction rate, which placed 14th among the 59 features.

**Potential impact:**

- The association between slower correction and higher mortality may be an epiphenomenon driven by underlying illness severity and treatment intensity rather than a direct causal link.
- Clinicians should avoid indiscriminately pursuing rapid correction and instead develop individualized treatment plans based on each patient’s overall clinical context.

## INTRODUCTION

Hyponatremia is the most common electrolyte abnormality among hospitalized patients and an independent predictor of mortality in various clinical settings [1–7]. The risk of mortality is particularly high in profound hyponatremia, which causes neurological symptoms requiring prompt intervention. However, overly aggressive correction can lead to a serious iatrogenic complication: osmotic demyelination syndrome (ODS) [8–13]. To prevent ODS, current guidelines recommend limiting the serum sodium (Na) correction rate to 4–8 mEq/l per 24 h, and clinicians have long practiced cautious treatment [14, 15].

However, this cautious approach has been challenged by recent large-scale observational studies. For example, a 25-year cohort study from two academic hospitals [16], an analysis using a multicenter intensive care unit database encompassing >200 hospitals [17], and a 16-year study from 21 community hospitals [18] indicated that slower correction was associated with increased mortality. This association was further supported by a systematic review, which noted that a slow correction group (<4–6 mEq/l/day) had 221 more in-hospital deaths per 1000 patients than a rapid correction group (≥8–10 mEq/l/day), and concluded that slow Na correction was associated with increased mortality risk [19]. Consequently, a wide discrepancy has emerged between existing guidelines and the latest evidence, sparking considerable controversy [20–25].

Although the association between correction rate and mortality has attracted attention, the relationship among correction rate, other clinical features, and in-hospital death remains unclear. The relationship between correction rate and mortality can be influenced by numerous factors, including comorbidities, overall condition, and treatment interventions [22]. However, these factors are difficult to analyze simultaneously using traditional methods, such as logistic regression, which has limitations in handling numerous variables and their complex interactions.

Therefore, we employed a machine-learning approach with three objectives: (i) to develop a model for predicting in-hospital mortality in profound hyponatremia and identify the most influential predictors; (ii) to evaluate the relative importance of the Na correction rate; and (iii) to clarify the interactions between the Na correction rate and other crucial features.

## MATERIALS AND METHODS

### Study design, setting, and participants

This single-center, retrospective cohort study included all adult patients (aged ≥18 years) with profound hyponatremia (serum Na ≤120 mEq/l) admitted to Chubu Rosai Hospital, a tertiary medical center in Japan, between January 2014 and December 2024. We excluded patients with (i) hyperglycemia (blood glucose ≥300 mg/dl), to exclude hyponatremia caused by osmotic shifts; (ii) death within 24 h of diagnosis; and (iii) no serum Na measurement at least 24 h after diagnosis. No formal a priori sample size calculation was performed; all eligible patients who met the inclusion criteria were included.

The study complied with the Declaration of Helsinki and was approved by the Chubu Rosai Hospital Clinical Research Ethics Committee (approval number: 202505-01), which waived the requirement for written informed consent, as the data were anonymized.

### Data collection

**Figure 1:**
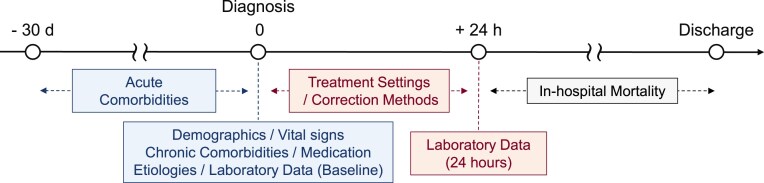
Data collection timeline. Baseline characteristics were collected at or before diagnosis. Information regarding postdiagnosis management was gathered from diagnosis up to 24 h later. The outcome of in-hospital mortality was assessed from 24 h post diagnosis until discharge.

Data were collected through automated extraction from electronic medical records and manual review of medical charts. To minimize information bias, one specialist initially extracted all variables, and other investigators independently verified the accuracy of the data. The features collected were divided into two categories (Fig. 1): (i) baseline characteristics at diagnosis, including demographics, vital signs, acute and chronic comorbidities, medications, etiologies, onset patterns of hyponatremia, and baseline laboratory data; and (ii) postdiagnosis management, including treatment setting, correction methods, and correction rate. Acute comorbidities were defined as diseases that developed within 30 days of diagnosis. For chronic comorbidities, we recorded the presence of distant metastasis from malignant tumors, the New York Heart Association (NYHA) functional classification for heart failure [26], the Child–Pugh classification for liver cirrhosis [27], and the Charlson comorbidity index (CCI) [28]. Acute hyponatremia was defined as a documented normal serum Na level (≥135 mEq/l) within 48 h before diagnosis [14, 15, 29].

Regarding postdiagnosis management, data within 24 h after diagnosis were extracted. For correction methods, intravenous fluid administration was classified into three categories based on electrolyte concentrations: (i) high-electrolyte (Na >154 mEq/l), (ii) normal-electrolyte (Na 130–154 mEq/l), and (iii) low-electrolyte (Na <130 mEq/l) solutions. The serum Na at 24 h after diagnosis (s[Na]₂₄) was estimated using linear interpolation: s[Na]₂₄ = Na_pre_ + [(Na_post_ – Na_pre_) × (24–T_pre_)/(T_post_–T_pre_)], where Na_pre_, Na_post_, T_pre_, and T_post_ represent the serum Na values and the times of measurement immediately before and after the 24-h mark, respectively [16, 29, 30]. The outcome was in-hospital mortality occurring ≥24 h after hyponatremia diagnosis.

### Statistical analysis

For descriptive statistics, patient characteristics were compared between the survivor and mortality groups. Continuous variables are presented as medians and interquartile ranges (IQRs) and compared using the Mann–Whitney U test. Categorical variables are presented as frequencies and percentages and compared using the chi-squared or Fisher’s exact tests. A two-tailed *P*-value <.05 was considered statistically significant.

### Feature selection and model development

The dataset was temporally split into a training set (January 2014–December 2022) and a held-out test set (January 2023–December 2024). Candidate features were screened within the training data for substantial missingness and multicollinearity; details of feature preprocessing are provided in the Supplementary Methods. To predict in-hospital mortality, we developed four machine-learning models: logistic regression, random forest (RF), extreme gradient boosting (XGBoost), and support vector machine (SVM). Feature selection was optimized separately for each model using recursive feature elimination with cross-validation (RFECV), which identifies the feature set with the best cross-validated area under the receiver operating characteristic curve (ROC-AUC) [31, 32].

### Model evaluation and interpretation

The performance of the final models was evaluated on the held-out test set. The primary performance metric was the ROC-AUC, for which 95% confidence intervals (CIs) were calculated via bootstrap resampling (*n* = 1000). We also calculated the sensitivity, specificity, and F1 score at the point of maximizing the Youden index. The Brier score was calculated to assess calibration [33]. Among the four machine-learning models, we identified the best model based on the highest ROC-AUC on the test data and interpreted it using SHapley Additive exPlanations (SHAP). In practical terms, SHAP estimates how much each variable contributes to the model’s mortality prediction for each patient. Feature importance was quantified as the mean absolute SHAP value, and SHAP summary and dependence plots were used to analyze the direction and magnitude of each feature’s effect.

To investigate the impact of slow Na correction on in-hospital mortality, SHAP dependence plots of key predictors were color-coded by 24-h Na correction rate category, and SHAP values were compared between correction rate categories after stratification by key predictors. The Na correction rate categories were defined as slow correction (<5 mEq/l/24 h) and nonslow correction (≥5 mEq/l/24 h), with alternative cutoffs of <4 and <6 mEq/l/24 h used in sensitivity analyses [14–16, 19, 29, 30]. Differences between groups were then evaluated using the Mann–Whitney U test, with a Bonferroni-corrected *P*- value <.05 considered statistically significant.

### Data collection: in-hospital deaths

To clarify the clinical circumstances surrounding in-hospital deaths, we collected additional data for two groups: (i) deaths included in the machine-learning analysis and (ii) deaths occurring within 24 h after diagnosis. These variables included time from hyponatremia diagnosis to death, serum Na level closest to death, primary cause of death, comfort-focused care, active attempt to correct serum Na, correction methods, and documented cerebral edema or brain herniation. Detailed definitions are provided in the footnotes of Supplementary Tables S7 and S8.

### Software

Descriptive statistics were performed using Stata version 19.0 MP (Stata Corp., College Station, TX). Feature selection, machine-learning model development, evaluation, and visualization were conducted using Python version 3.10 (Python Software Foundation).

## RESULTS

### Patient characteristics

During the study period, 636 patients with profound hyponatremia were hospitalized. After applying the eligibility criteria, 546 patients were included in the analysis (Supplementary Fig. S1). The median age was 77 years (IQR, 68–84), and the median baseline serum Na level was 118 mEq/l (IQR, 115–119) (Table 1). Overall, 99 patients (18%) died in the hospital, and mortality was lower in patients with lower baseline serum Na levels: 11.6% in the ≤110 mEq/l group, 13.2% in the 111–115 mEq/l group, and 20.4% in the 116–120 mEq/l group (Supplementary Table S1). The median 24-h serum Na correction rate was 4 mEq/l/24 h (IQR, 2–7) (Supplementary Fig. S2).

**Table 1:** Characteristics of all patients, in-hospital survivor group, and the mortality group.

	All*n* = 546	Survivor group*n* = 447	Mortality group *n* = 99
Baseline characteristics at diagnosis			
Demographics			
Age, year, median (IQR)	77 (68–84)	77 (67–84)	78 (71–85)
Female, *n* (%)	238 (44)	200 (45)	38 (38)
Body mass index, kg/m^2^, median (IQR)	19.4 (17.2–22.7)	19.7 (17.4–22.9)	18.7 (16.3–21.3)
Vital signs and support			
Systolic BP, mm Hg, median (IQR)	131 (112–151)	133 (114–154)	122 (103–138)
Diastolic BP, mm Hg, median (IQR)	73 (63–88)	75 (65–89)	66 (54–78)
Vasopressor use, *n* (%)	21 (4)	11 (2)	10 (10)
Oxygen administration, *n* (%)	88 (16)	59 (13)	29 (29)
Mechanical ventilation, *n* (%)	5 (0.9)	2 (0.4)	3 (3)
Pre-admission status and chronic comorbidities			
Community-onset hyponatremia, *n* (%)	316 (58)	277 (62)	39 (39)
Acute hyponatremia	2 (0.4)	2 (0.4)	0 (0)
CCI, median (IQR)	2 (1–4)	2 (1–3)	4 (2–6)
Chronic heart failure, *n* (%)	109 (20)	78 (17)	31 (31)
NYHA I–II, *n* (%)	13 (2)	12 (3)	1 (1)
NYHA III–IV, *n* (%)	14 (3)	7 (2)	7 (7)
NYHA unknown, *n* (%)	82 (15)	59 (13)	23 (23)
Chronic kidney disease, *n* (%)	111 (20)	88 (20)	23 (23)
Maintenance dialysis, *n* (%)	6 (1)	6 (1)	0 (0)
Liver cirrhosis, *n* (%)	26 (5)	15 (3)	11 (11)
Child–Pugh A, *n* (%)	6 (1)	5 (1)	1 (1)
Child–Pugh B, *n* (%)	6 (1)	4 (0.9)	2 (2)
Child–Pugh C, *n* (%)	14 (3)	6 (1)	8 (8)
Solid tumor without metastasis, *n* (%)	60 (11)	51 (11)	9 (9)
Metastatic malignant tumor, *n* (%)	93 (17)	52 (12)	41 (41)
Pre-admission Medications, *n* (%)			
Loop diuretics	94 (17)	68 (15)	26 (26)
Thiazide diuretics	59 (11)	57 (13)	2 (2)
SSRI/SNRI	17 (3)	17 (4)	0 (0)
NSAIDs	69 (13)	54 (12)	15 (15)
Opioids	24 (4)	9 (2)	15 (15)
Acute comorbidities in 30 days, *n* (%)			
Pneumonia	94 (17)	67 (15)	27 (27)
Urinary tract infection	39 (7)	34 (8)	5 (5)
Skin and soft-tissue infection	10 (2)	9 (2)	1 (1)
Acute heart failure	46 (8)	34 (8)	12 (12)
Acute myocardial infarction	2 (0.4)	1 (0.2)	1 (1)
Stroke	16 (3)	14 (3)	2 (2)
Acute kidney injury	90 (16)	68 (15)	22 (22)
Fracture	36 (7)	34 (8)	2 (2)
Etiology of hyponatremia, *n* (%)			
Primary polydipsia	34 (6)	33 (7)	1 (1)
Hypovolemia	68 (12)	54 (12)	14 (14)
SIAD	342 (63)	277 (62)	65 (66)
Drug-related	81 (15)	79 (18)	2 (2)
Adrenal insufficiency	13 (2)	9 (2)	4 (4)
Unidentified cause	68 (12)	54 (12)	14 (14)
Laboratory data, median (IQR)			
Serum Na, mEq/l (baseline)	118 (115–119)	117 (114–119)	119 (116–120)
Serum K, mEq/l	4.3 (3.9–4.9)	4.3 (3.8–4.8)	4.5 (3.9–5.2)
Albumin, g/dl	3.2 (2.6–3.8)	3.4 (2.8–4.0)	2.5 (2.1–3.1)
eGFR, ml/min/1.73 m^2^	79 (49–111)	79 (49–110)	76 (47–114)
CRP, mg/dl	1.4 (0.3–6.4)	1.0 (0.2–5.1)	4.9 (1.6–9.6)
Glucose, mg/dl	124 (104–152)	126 (106–152)	118 (95–152)
White blood cell count, ×10^2^/μl	81 (56–108)	78 (56–104)	96 (61–139)
Hemoglobin, g/dl	11.1 (9.9–12.6)	11.3 (10.0–12.8)	10.0 (9.1–11.6)
Platelet count, ×10^4^/μl	21.3 (15.7–28.0)	21.5 (16.4–28.0)	18.7 (12.1–29.0)
Urinary Na, mEq/l	59 (29–90)	61 (30–90)	45 (20–82)
Urinary K, mEq/l	28 (18–41)	28 (18–41)	28 (20–38)
Postdiagnosis management			
Treatment settings, *n* (%)			
Intensive care unit	119 (22)	104 (23)	15 (15)
Department of internal medicine	321 (59)	258 (58)	63 (64)
Department of surgery	106 (19)	85 (19)	21 (21)
Correction methods and monitoring			
High-electrolyte solution (bolus), *n* (%)	73 (13)	66 (15)	7 (7)
High-electrolyte solution (continuous), *n* (%)	213 (39)	190 (43)	23 (23)
Normal-electrolyte solution, *n* (%)	257 (47)	193 (43)	64 (65)
Low-electrolyte solution, *n* (%)	175 (32)	160 (36)	15 (15)
Desmopressin, *n* (%)	79 (14)	76 (17)	3 (3)
Loop diuretics, *n* (%)	72 (13)	55 (12)	17 (17)
Vaptans, *n* (%)	15 (3)	9 (2)	6 (6)
Serum Na measurements, median (IQR)	3 (2–6)	3 (2–6)	2 (1–4)
Laboratory data after 24 h			
Serum Na, mEq/l, median (IQR)	121 (119–123)	121 (119–124)	121 (119–122)
ΔSerum Na, mEq/l, median (IQR)	4 (2–7)	5 (2–7)	2 (1–5)

BP, blood pressure; eGFR, estimated glomerular filtration rate; K, potassium; Na, sodium; NSAIDs, nonsteroidal anti-inflammatory drugs; SIAD, syndrome of inappropriate antidiuresis; SSRI/SNRI, selective serotonin reuptake inhibitor/serotonin-norepinephrine reuptake inhibitor; ΔSerum Na, serum sodium correction rate.

### Comparison between the survivor and mortality groups

Compared with the survivor group, the mortality group had a higher CCI and a higher prevalence of severe heart failure (NYHA III–IV: 7% vs 2%), severe liver cirrhosis (Child–Pugh C: 8% vs 1%), and metastatic malignant tumors (41% vs 12%). Regarding baseline laboratory values, the mortality group had higher serum Na (median, 119 vs 117 mEq/l) and C-reactive protein (CRP) levels (median, 4.9 vs 1.0 mg/dl), but lower albumin levels (median, 2.5 vs 3.4 g/dl). Regarding treatment, normal-electrolyte solutions were used more frequently in the mortality group (65% vs 43%), whereas high-electrolyte and low-electrolyte solutions were used less frequently. The mortality group had fewer Na measurements (median, 2 vs 3) and a slower correction rate (median, 2 vs 5 mEq/l/24 h). No patient developed clinically evident ODS.

### Feature selection, model development, and performance

**Figure 2:**
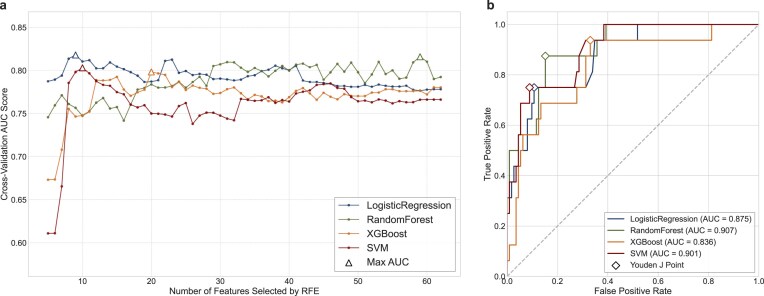
Feature selection process and predictive performance of the machine-learning models. (a) The optimal number of features for each model was determined by RFECV on the training data. Performance was measured by the cross-validated AUC. The triangular markers indicate the feature set that yielded the maximum AUC. (b) ROC curves show the performance of the final, optimized models on the independent test data. The diamond-shaped markers represent the Youden J point, where the Youden index is maximized.

Of the 66 features listed in Table 1, 62 remained after excluding variables with substantial missingness or high correlation. The number of features selected using RFECV was 9 for logistic regression, 59 for RF, 20 for XGBoost, and 10 for SVM (Fig. 2a and Supplementary Fig. S4). Model performance was evaluated in the independent test set (Table 2). The RF model showed the highest discrimination, with an AUC of 0.907 (95% CI, 0.832–0.965) (Fig. 2b), and the lowest Brier score of 0.087. Baseline models using only features available at diagnosis also showed high discrimination (AUC, 0.868; 95% CI, 0.786–0.937) (Supplementary Table S4).

**Table 2:** Predictive performance of the machine-learning models.

Model	No. of features	ROC-AUC (95% CI)	Youden index	Optimal threshold	Sensitivity	Specificity	F1 score	Brier score
Logistic regression	9	0.875 (0.789–0.950)	0.643	0.714	0.750	0.893	0.564	0.170
RF	59	0.907 (0.832–0.965)	0.723	0.337	0.875	0.848	0.565	0.087
XGBoost	20	0.836 (0.709–0.927)	0.607	0.069	0.938	0.670	0.418	0.100
SVM	10	0.901 (0.828–0.962)	0.661	0.749	0.750	0.911	0.595	0.159

No., number.

### Interpretation of the best-performing model

**Figure 3:**
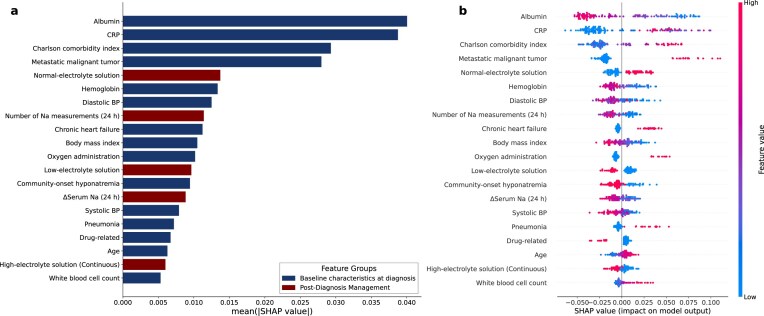
SHAP analysis of the RF model. SHAP values represent the contribution of each feature to the model prediction. (a) The feature importance plot ranks features by their mean absolute SHAP value. Bars are color-coded by feature group: baseline characteristics at diagnosis and postdiagnosis management. (b) The SHAP summary plot shows the distribution of impacts for each feature. Each point corresponds to a single patient, with color indicating the feature value. The horizontal axis represents the SHAP value, indicating the direction and magnitude of impact of the feature on the prediction. BP, blood pressure; K, potassium; Na, sodium; ΔSerum Na, serum sodium correction rate.

**Figure 4:**
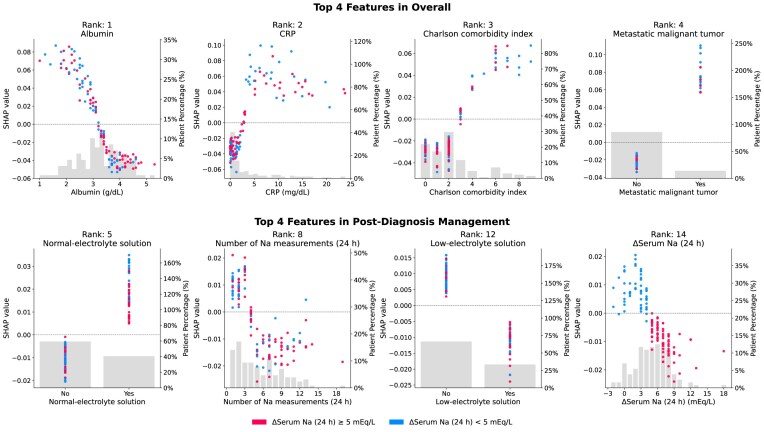
SHAP dependence plots colored by serum Na correction rates. The plots illustrate the impact of the top four predictors in the ‘Overall’ category (top row) and the ‘Postdiagnosis management’ category (bottom row). Each dot represents a single patient, plotting the value of the feature (*x*-axis) against its corresponding SHAP value (left *y*-axis). The background gray histograms show the patients distribution (right *y*-axis). Points are color-coded by the 24-h serum Na correction rate: <5 mEq/l and ≥5 mEq/l. Na, sodium; ΔSerum Na, serum sodium correction rate.

SHAP analysis of the RF model showed that the most important predictors of in-hospital mortality were baseline severity markers: serum albumin, CRP, CCI, and metastatic malignant tumor (Fig. 3a). Low albumin, high CRP, high CCI, and the presence of metastatic malignancy were associated with higher SHAP values (Figs 3b and 4 and Supplementary Fig. S5). Among postdiagnosis management features, normal-electrolyte solutions use (rank 5), the number of Na measurements (rank 8), and low-electrolyte solutions use (rank 12) ranked highly, followed by the 24-h Na correction rate (rank 14). The normal-electrolyte solutions use and fewer Na measurements were associated with higher SHAP values, whereas the low-electrolyte solutions use and faster correction were associated with lower SHAP values (Figs 3b and 4 and Supplementary Fig. S5).

The SHAP values of top-ranked baseline features did not differ significantly across correction rate categories (all *P*-values >.05) (Table 3 and Fig. 4). However, for normal-electrolyte solution use, the mean SHAP value was 1.84-fold higher in the slow correction group, with consistent findings in sensitivity analyses (Supplementary Table S5). Compared with the low-electrolyte solution group, the normal-electrolyte solution group had a higher CCI and a higher prevalence of Child–Pugh C liver cirrhosis and metastatic malignancy (Supplementary Table S6).

**Table 3:** Subgroup analysis results of mean SHAP values of key features.

Feature	Subgroup	ΔSerum Na <5 mEq/l	ΔSerum Na ≥5 mEq/l	SHAP ratio	Uncorrected*P*-value	Corrected*P*-value
Albumin	≥3.0 g/dl	−0.0252	−0.0248	1.02	.54	>.99
	<3.0 g/dl	0.0572	0.0551	1.04	.83	>.99
CRP	≥1.0 mg/dl	0.0247	0.0194	1.27	.36	>.99
	<1.0 mg/dl	−0.0372	−0.0323	1.15	.06	.98
CCI	≥3 points	0.039	0.026	1.5	.19	>.99
	≤2 points	−0.0276	−0.0278	0.99	.67	>.99
Metastatic malignant tumor	Yes	0.0829	0.0689	1.2	.13	>.99
	No	−0.021	−0.0191	1.1	.03	.45
Normal-electrolyte solution	Yes	0.026	0.0141	1.84	<.001	<.001
	No	−0.0114	−0.0092	1.24	.1	>.99
Serum Na measurements (24 h)	≥3 times	−0.0077	−0.0085	0.91	.66	>.99
	≤2 times	0.0093	0.0096	0.97	.9	>.99
Low-electrolyte solution	Yes	−0.0117	−0.0111	1.05	.27	>.99
	No	0.0093	0.0083	1.12	.24	>.99
ΔSerum Na (24 h)	≥5 mEq/l	N/A	−0.0095	N/A	N/A	N/A
	<5 mEq/l	0.0077	N/A	N/A	N/A	N/A

Na, sodium; N/A, not applicable; ΔSerum Na, serum sodium correction rate.

### Clinical course and causes of in-hospital deaths

Among the 99 deaths included in the analytic cohort, the median time from hyponatremia diagnosis to death was 18 days (IQR, 10–40), and the median serum Na closest to death was 134 mEq/l (IQR, 128.5–141). The most common causes of death were malignant tumors (40%), infections (24%), and circulatory diseases (14%); no deaths were attributed primarily to hyponatremia based on clinical documentation (Supplementary Table S7). Comfort-focused care was documented in 48 patients (48%), and active serum Na correction was not documented in 44 patients (44%). No documented cerebral edema or brain herniation was identified; for both outcomes, 9 patients (9%) were evaluated and had no documented findings, whereas 90 (91%) were not evaluated.

Among patients excluded from the machine-learning analysis, 20 died within 24 h after diagnosis (Supplementary Table S8). The most common causes of death were malignant tumors (45%), circulatory diseases (20%), infections (15%), and gastrointestinal diseases (15%); none were attributed primarily to hyponatremia. Comfort-focused care was documented in 17 patients (85%), and active serum Na correction was not documented in 18 patients (90%). No documented cerebral edema or brain herniation was identified; for both outcomes, 7 patients (35%) were evaluated and had no documented findings, whereas 13 (65%) were not evaluated.

## DISCUSSION

In this study, we developed four machine-learning models to predict in-hospital mortality in patients with profound hyponatremia, and the RF model demonstrated the best predictive performance. SHAP analysis identified baseline characteristics at diagnosis as the most influential predictors. Among postdiagnosis features, treatment processes such as fluid selection and monitoring frequency had a greater impact than the correction rate. The predictive contributions of fluid selection varied with correction rate, whereas top-ranked baseline characteristics were consistent regardless of whether the correction was slow or not.

To our knowledge, this is the first study to use machine learning to predict and interpret mortality in patients with profound hyponatremia. Despite the heterogeneous etiologies and comorbidities of patients with profound hyponatremia, we collected numerous features through chart review and developed predictive models with excellent performance. The best-performing model was RF, an ensemble of decision trees constructed via a dual randomization process [34, 35]. This process helps prevent overfitting even with high-dimensional data and maintains high generalizability [36, 37], but introduces a “black box” problem due to the opacity of its predictive logic. To address this limitation, we used SHAP, which visualizes the model’s decision-making process by quantifying each feature’s contribution [38, 39], enabling us to rank a wide range of features beyond the scope of conventional studies.

SHAP analysis revealed that baseline patient characteristics at diagnosis were the most important predictors of in-hospital mortality. Albumin, CRP, CCI, and metastatic malignancy are well-established indicators of poor prognosis, and our findings reaffirm their critical importance [40–42]. A novel finding of this study is that the predictive impact of the top-ranked features was consistent regardless of the correction rate. This consistency is clinically meaningful; although hyponatremia has been suggested to increase mortality risk through oxidative and osmotic stress [19, 43, 44], our findings suggest that early differences in correction rates do not substantially influence mortality risk associated with underlying disease. Moreover, models using only baseline features demonstrated performance comparable to those incorporating treatment variables, suggesting that prognosis was largely determined at the time of diagnosis. Additional descriptive analyses showed lower mortality among patients with extremely low baseline serum Na levels; moreover, serum Na levels before death were normal or only mildly decreased in many cases, and no deaths were attributed primarily to hyponatremia. These findings support the hypothesis that patients die from the underlying diseases rather than from hyponatremia itself [22, 42, 45].

Furthermore, fluid choice and monitoring frequency were stronger predictors of mortality than the Na correction rate itself. These features may serve as surrogate markers for treatment intensity and responsiveness. For example, in frail or end-of-life patients, clinicians often withhold aggressive correction [22, 45]; thus, less frequent monitoring and supportive care with normal-electrolyte solutions may indicate lower treatment intensity for patients with poor prognosis. Consistent with this interpretation, comfort-focused care or the absence of an active attempt to correct serum Na was observed in a substantial proportion of deaths. In addition, the association between normal-electrolyte solution use and mortality was stronger when correction was slow. The group treated with normal-electrolyte solutions included more patients with advanced cancer or liver cirrhosis. These life-threatening conditions often lead to treatment resistance due to persistent antidiuretic hormone secretion [22]. Conversely, low-electrolyte solution use was associated with survival, as these solutions are typically administered to prevent overcorrection in patients who have reversible etiologies with a favorable prognosis, such as primary polydipsia, drug-induced hyponatremia, or transient stress [29].

Our analysis identified multiple key predictors of in-hospital mortality beyond the correction rate, suggesting that the association between slow Na correction and high mortality may be an epiphenomenon rather than a direct causal link. Most existing observational studies have defined exposure using “achieved correction rates” rather than “intended correction rates.” [16–19] This distinction is critical because the achieved correction rate is influenced by both treatment intensity and responsiveness; reduced treatment intensity or increased treatment resistance in patients with poor prognosis may result in slower correction, raising the possibility of reverse causation. Therefore, clinicians should not indiscriminately aim for rapid correction but instead develop individualized treatment plans that optimize each patient’s risk–benefit profile, considering management of underlying diseases, the severity of hyponatremia symptoms, tolerance of fluid therapy, and patients’ goals of care.

This study has several limitations. First, to accurately assess the impact of the 24-h Na correction rate, we intentionally excluded patients who died within 24 h from the machine-learning analysis, which may have underestimated mortality and biased the assessment of predictive factors. Second, the correction rate in our cohort was relatively slow, resulting in insufficient power to assess the risk of ODS. However, the high proportion of patients with slow correction (42% with <4 mEq/l/24 h) was advantageous for examining the association between slower correction and poor outcomes. Third, although manual chart review provides detailed clinical information, the retrospective observational design leaves our findings susceptible to unrecorded or incompletely documented features and residual confounding. In addition, assessment of cerebral edema and brain herniation was also limited because many patients did not undergo neuroimaging or autopsy; therefore, undiagnosed cerebral edema or herniation may have been missed. Fourth, although SHAP analysis has been widely used to interpret clinical prediction models [33, 46, 47], the associations identified are correlational and do not establish causation; therefore, our findings should be interpreted as hypothesis-generating. Finally, the single-center design limits generalizability. Although we used a temporal holdout design for internal validation, the model has not been externally validated across different institutions, treatment practices, or patient populations. Future multicenter studies using harmonized data collection are needed to validate and refine our findings.

In conclusion, we developed machine-learning models to predict in-hospital mortality in patients with profound hyponatremia. SHAP analysis revealed that baseline patient characteristics, fluid choice, and monitoring frequency were stronger predictors of mortality than the Na correction rate. Prospective studies with external validation are needed to confirm the findings of this study.

## Supplementary Material

sfag219_Supplemental_File

## Data Availability

The analytical datasets and Python and Stata programs used in this study are available upon reasonable request to the corresponding author, provided the authors approve the specified purpose.
