# Supplementary material for: Key predictors of mortality in profound hyponatremia beyond the correction rate
Source: Clin Kidney J. 2026 Jun 30;19(8):sfag219. doi: 10.1093/ckj/sfag219 (PMC13430268; doi:10.1093/ckj/sfag219)
Supplement: sfag219_Supplemental_File [file sfag219_supplemental_file.pdf]

Supplementary Material

Key predictors of in-hospital mortality in profound hyponatremia beyond the sodium correction rate: an interpretable machine learning approach

Supplemental Methods

Supplemental Tables

Supplementary Table S1. In-hospital mortality according to baseline serum sodium category

Supplementary Table S2. Characteristics of all patients and the training and test sets

Supplementary Table S3. Hyperparameter tuning for each machine learning classifier

Supplementary Table S4. Predictive performance of the machine learning models (baseline models)

Supplementary Table S5. Subgroup analysis of mean SHAP values for key features using alternative cutoffs for the sodium correction rate

Supplementary Table S6. Baseline characteristics by hyponatremia management strategy

Supplementary Table S7. Clinical course among in-hospital deaths included in the analytic cohort

Supplementary Table S8. Clinical course among patients who died within 24 hours after hyponatremia diagnosis

Supplemental Figures

Supplementary Figure S1. Patient selection flowchart

Supplementary Figure S2. Distribution of 24-hour changes in the serum sodium level

Supplementary Figure S3. Spearman correlation matrix (variables with |r|>0.5)

Supplementary Figure S4. Features selected by each machine learning classifier

Supplementary Figure S5. SHAP dependence plots for all selected features (full model)

Supplementary Figure S6. SHAP dependence plots for all selected features (baseline model)

Supplemental Methods

The dataset was split using a temporal holdout approach: patients diagnosed between January 2014 and December 2022 were assigned to the training set, and those diagnosed between January 2023 and December 2024 to the held-out test set. All 66 candidate features listed in Table 1 were screened within the training data. Variables with ≥5% missing values in the training set were excluded; accordingly, urine Na and urine K were excluded because they had missing values in 78 patients (19%). Among the remaining 64 variables, two pairs exhibited high correlation (Spearman $|r|>0.8$): chronic heart failure and unknown NYHA functional classification ($r=0.878$), and drug-related hyponatremia and thiazide diuretics ($r=0.851$) (Supplementary Figure S3). From each pair, the variable with the lower univariate ROC-AUC for in-hospital mortality was removed (unknown NYHA functional classification and thiazide diuretics), leaving 62 variables for model development.

Among these 62 variables, only body mass index had missing values (6 cases, 1%), which were imputed using k-nearest neighbors imputation with $k=5$. Continuous variables were standardized, and the Yeo–Johnson transformation was applied to approximate a normal distribution. The synthetic minority oversampling technique (SMOTE) was applied only within the training folds during cross-validation to address class imbalance. Hyperparameters were tuned using grid search with stratified five-fold time-series cross-validation. The hyperparameter search spaces and selected parameters are shown in Supplementary Table S3. As a supplementary analysis, we developed baseline models using only features available at diagnosis, applying the identical pipeline as the primary analysis.

Supplementary Table S1. In-hospital mortality according to baseline serum sodium category

Baseline Na category	Total patients	In-hospital deaths	Mortality
Overall	546	99	18.1%
≤110 mEq/L	43	5	11.6%
111–115 mEq/L	121	16	13.2%
116–120 mEq/L	382	78	20.4%

Abbreviation: Na, sodium.

Supplementary Table S2. Characteristics of all patients and the training and test sets

	All<br>n=546	Training set<br>n=418	Test set<br>n=128
**<u>Baseline Characteristics at Diagnosis</u>**			
**Demographics**			
Age, yr, median (IQR)	77 (68–84)	77 (68–84)	76 (68–85)
Female, n (%)	238 (44)	182 (44)	56 (44)
Body mass index, kg/m², median (IQR)	19.4 (17.2–22.7)	19.6 (17.3–22.9)	19.4 (16.9–22.1)
**Vital Signs and Support**			
Systolic BP, mm Hg, median (IQR)	131 (112–151)	130 (111–148)	137 (117–156)
Diastolic BP, mm Hg, median (IQR)	73 (63–88)	72 (62–86)	77 (66–92)
Vasopressor use, n (%)	21 (4)	20 (5)	1 (0.8)
Oxygen administration, n (%)	88 (16)	76 (18)	12 (9)
Mechanical ventilation, n (%)	5 (0.9)	5 (1)	0 (0)
**Pre-admission Status and Chronic Comorbidities**			
Community-onset hyponatremia, n (%)	316 (58)	219 (52)	97 (76)
Acute hyponatremia	2 (0.4)	0 (0)	2 (2)
Charlson comorbidity index, median (IQR)	2 (1–4)	2 (1–4)	2 (1–3)
Chronic heart failure, n (%)	109 (20)	77 (18)	32 (25)
NYHA I-II, n (%)	13 (2)	6 (1)	7 (5)
NYHA III-IV, n (%)	14 (3)	9 (2)	5 (4)
NYHA unknown, n (%)	82 (15)	62 (15)	20 (16)
Chronic kidney disease, n (%)	111 (20)	84 (20)	27 (21)
Maintenance dialysis, n (%)	6 (1)	6 (1)	0 (0)
Liver cirrhosis, n (%)	26 (5)	19 (5)	7 (5)
Child–Pugh A, n (%)	6 (1)	6 (1)	0 (0)
Child–Pugh B, n (%)	6 (1)	3 (0.7)	3 (2)
Child–Pugh C, n (%)	14 (3)	10 (2)	4 (3)
Solid tumor without metastasis, n (%)	60 (11)	43 (10)	17 (13)
Metastatic malignant tumor, n (%)	93 (17)	75 (18)	18 (14)
**Pre-admission Medications, n (%)**			
Loop diuretics	94 (17)	75 (18)	19 (15)
Thiazide diuretics	59 (11)	50 (12)	9 (7)
SSRI/SNRI	17 (3)	12 (3)	5 (4)
NSAIDs	69 (13)	59 (14)	10 (8)
Opioids	24 (4)	21 (5)	3 (2)

	All<br>n=546	Training set<br>n=418	Test set<br>n=128
**Acute Comorbidities in 30 Days, n (%)**			
Pneumonia	94 (17)	78 (19)	16 (13)
Urinary tract infection	39 (7)	27 (6)	12 (9)
Skin and soft-tissue infection	10 (2)	9 (2)	1 (0.8)
Acute heart failure	46 (8)	34 (8)	12 (9)
Acute myocardial infarction	2 (0.4)	1 (0.2)	1 (0.8)
Stroke	16 (3)	12 (3)	4 (3)
Acute kidney injury	90 (16)	66 (16)	24 (19)
Fracture	36 (7)	24 (6)	12 (9)
**Etiology of hyponatremia, n (%)**			
Primary polydipsia	34 (6)	24 (6)	10 (8)
Hypovolemia	68 (12)	51 (12)	17 (13)
SIAD	342 (63)	268 (64)	74 (58)
Drug-related	81 (15)	66 (16)	15 (12)
Adrenal insufficiency	13 (2)	9 (2)	4 (3)
Unidentified cause	68 (12)	57 (14)	11 (9)
**Laboratory Data, median (IQR)**			
Serum Na, mEq/L (baseline)	118 (115–119)	118 (115–119)	118 (114–119)
Serum K, mEq/L	4.3 (3.9–4.9)	4.3 (3.9–4.9)	4.3 (3.8–4.8)
Albumin, g/dL	3.2 (2.6–3.8)	3.2 (2.6–3.8)	3.3 (2.6–3.9)
eGFR, mL/min/1.73 m^2	79 (49–111)	78 (48–111)	80 (53–112)
CRP, mg/dL	1.4 (0.3–6.4)	1.5 (0.3–6.7)	1.4 (0.2–5.7)
Glucose, mg/dL	124 (104–152)	123 (102–152)	128 (112–156)
White blood cell count, $\times 10^2/\mu L$	81 (56–108)	82 (55–108)	79 (61–109)
Hemoglobin, g/dL	11.1 (9.9–12.6)	11.1 (9.8–12.4)	11.5 (10.0–12.8)
Platelet count, $\times 10^4/\mu L$	21.3 (15.7–28.0)	21.3 (15.5–28.0)	21.3 (16.3–28.1)
Urinary Na, mEq/L	59 (29–90)	60 (29–90)	55 (29–89)
Urinary K, mEq/L	28 (18–41)	27 (18–39)	31 (19–45)
**Post-Diagnosis Management**			
**Treatment Settings, n (%)**			
Intensive care unit	119 (22)	74 (18)	45 (35)
Department of Internal Medicine	321 (59)	258 (62)	63 (49)
Department of Surgery	106 (19)	86 (21)	20 (16)
**Correction Methods and Monitoring**			
High-electrolyte solution (bolus), n (%)	73 (13)	34 (8)	39 (30)
High-electrolyte solution (continuous), n (%)	213 (39)	157 (38)	56 (44)

	All n=546	Training set n=418	Test set n=128
Normal-electrolyte solution, n (%)	257 (47)	205 (49)	52 (41)
Low-electrolyte solution, n (%)	175 (32)	132 (32)	43 (34)
Desmopressin, n (%)	79 (14)	55 (13)	24 (19)
Loop diuretics, n (%)	72 (13)	58 (14)	14 (11)
Vaptans, n (%)	15 (3)	13 (3)	2 (2)
Serum Na measurements, median (IQR)	3 (2–6)	3 (2–5)	4 (2–8)
**Laboratory Data after 24 h**			
Serum Na, mEq/L, median (IQR)	121 (119–123)	121 (119–123)	122 (119–124)
ΔSerum Na, mEq/L, median (IQR)	4 (2–7)	4 (2–6)	5 (3–7)

Abbreviations: BP, blood pressure; CRP, C-reactive protein; eGFR, estimated glomerular filtration rate; IQR, interquartile range; K, potassium; Na, sodium; NSAIDs, non-steroidal anti-inflammatory drugs; NYHA, New York Heart Association; SIAD, syndrome of inappropriate antidiuresis; SSRI/SNRI, selective serotonin reuptake inhibitor/serotonin-norepinephrine reuptake inhibitor; ΔSerum Na, serum sodium correction rate.

Supplementary Table S3. Hyperparameter tuning for each machine learning classifier

Models	Hyperparameter Search Space	Selected Optimal Parameters
**Logistic Regression**	Grid 1 (ElasticNet)<br>solver: ['saga']<br>penalty: ['elasticnet']<br>C: [0.01, 0.1, 1, 10, 100]<br>l1_ratio: [0, 1]<br><br>Grid 2 (L1/L2)<br>solver: ['liblinear']<br>penalty: ['l1', 'l2']<br>C: [0.01, 0.1, 1, 10, 100]	solver: saga<br>penalty: elasticnet<br>C: 1<br>l1_ratio: 0
**Random Forest**	n_estimators: [100, 200]<br>max_depth: [10, 20, None]<br>min_samples_leaf: [1, 2]<br>max_features: ['sqrt', 'log2']	n_estimators: 200<br>max_depth: 20<br>min_samples_leaf: 2<br>max_features: log2
**XGBoost**	n_estimators: [100, 200]<br>max_depth: [3, 6, 9]<br>learning_rate: [0.01, 0.1, 0.2]<br>subsample: [0.8]<br>colsample_bytree: [0.8]	n_estimators: 100<br>max_depth: 6<br>learning_rate: 0.2<br>subsample: 0.8<br>colsample_bytree: 0.8
**Support Vector Machine**	Grid 1 (linear kernel)<br>kernel: ['linear']<br>C: [0.01, 0.1, 1, 10, 100]<br>Grid 2 (rbf kernel)<br>kernel: ['rbf']<br>C: [0.01, 0.1, 1, 10, 100]<br>gamma: ['scale', 'auto']	kernel: linear<br>C: 10

The hyperparameter names listed in this table correspond to the argument names used in the Python libraries employed for model implementation: scikit-learn (for logistic regression, random forest, and support vector machine models) and XGBoost (for the XGBoost model).

Abbreviations: XGBoost, eXtreme Gradient Boosting

Supplementary Table S4. Predictive performance of the machine learning models (baseline models)

Model	No. of Features	ROC-AUC (95% CI)	Youden Index	Optimal Threshold	Sensitivity	Specificity	F1 Score	Brier Score
**Logistic Regression**	14	0.868 (0.781–0.939)	0.616	0.729	0.750	0.866	0.524	0.172
**Random Forest**	26	0.868 (0.786–0.937)	0.625	0.322	0.813	0.813	0.490	0.099
**XGBoost**	16	0.824 (0.720–0.910)	0.554	0.133	0.813	0.741	0.421	0.120
**Support Vector Machine**	26	0.855 (0.755–0.933)	0.589	0.531	0.813	0.777	0.453	0.162

Abbreviations: CI, confidence interval; ROC-AUC, area under the receiver operating characteristic curve; XGBoost, extreme gradient boosting; no., number.

Supplementary Table S5. Subgroup analysis of mean SHAP values for key features using alternative cutoffs for sodium correction rate

A) Cutoff value: 4 mEq/L

Feature	Subgroup	ΔSerum Na <4 mEq/L	ΔSerum Na ≥4 mEq/L	SHAP Ratio	Uncorrected *p*-value	Corrected *p*-value
**Albumin**	≥3.0 g/dL	-0.0235	-0.0254	0.93	0.73	>0.99
	<3.0 g/dL	0.0588	0.0544	1.08	0.5	>0.99
**CRP**	≥1.0 mg/dL	0.0257	0.02	1.29	0.32	>0.99
	<1.0 mg/dL	-0.0368	-0.0329	1.12	0.17	>0.99
**Charlson comorbidity index**	≥3 points	0.0364	0.0307	1.19	0.75	>0.99
	≤2 points	-0.0285	-0.0274	1.04	0.76	>0.99
**Metastatic malignant tumor**	Yes	0.0908	0.0708	1.28	0.007	0.11
	No	-0.0215	-0.0191	1.13	0.009	0.14
**Normal-electrolyte solution**	Yes	0.0269	0.0147	1.83	<0.001	<0.001
	No	-0.0121	-0.0094	1.29	0.06	0.93
**Serum Na measurements (24 h)**	≥3 times	-0.0057	-0.0088	0.65	0.22	>0.99
	≤2 times	0.009	0.0099	0.91	0.79	>0.99
**Low-electrolyte solution**	Yes	-0.0123	-0.0111	1.11	0.26	>0.99
	No	0.0097	0.0083	1.17	0.07	>0.99
**ΔSerum Na (24 h)**	≥4 mEq/L	N/A	-0.0074	N/A	N/A	N/A
	<4 mEq/L	0.0092	N/A	N/A	N/A	N/A

B) Cutoff value: 6 mEq/L

Feature	Subgroup	ΔSerum Na <6 mEq/L	ΔSerum Na ≥6 mEq/L	SHAP Ratio	Uncorrected *p*-value	Corrected *p*-value
**Albumin**	≥3.0 g/dL	-0.0218	-0.0277	0.79	0.82	>0.99
	<3.0 g/dL	0.0581	0.0527	1.10	0.42	>0.99
**CRP**	≥1.0 mg/dL	0.0239	0.0190	1.26	0.44	>0.99
	<1.0 mg/dL	-0.0375	-0.0313	1.20	0.009	0.14
**Charlson comorbidity index**	≥3 points	0.0372	0.0262	1.42	0.24	>0.99
	≤2 points	-0.0282	-0.0272	1.04	0.54	>0.99
**Metastatic malignant tumor**	Yes	0.0820	0.0684	1.20	0.12	>0.99
	No	-0.0209	-0.0188	1.11	0.004	0.06
**Normal-electrolyte solution**	Yes	0.0231	0.0140	1.65	<0.001	0.003
	No	-0.0112	-0.0089	1.26	0.054	0.86
**Serum Na measurements (24 h)**	≥3 times	-0.0075	-0.0090	0.83	0.42	>0.99
	≤2 times	0.0090	0.0102	0.88	0.67	>0.99
**Low-electrolyte solution**	Yes	-0.0133	-0.0100	1.33	0.02	0.28
	No	0.0089	0.0087	1.02	>0.99	>0.99
**ΔSerum Na (24 h)**	≥6 mEq/L	N/A	-0.0105	N/A	N/A	N/A
	<6 mEq/L	0.0049	N/A	N/A	N/A	N/A

Abbreviations: CRP, C-reactive protein; Na, sodium; N/A, not applicable; SHAP, SHapley Additive exPlanations; ΔSerum Na, serum sodium correction rate.

Supplementary Table S6. Baseline characteristics by hyponatremia management strategy

	Normal electrolyte solution only n=206	Low electrolyte solution only n=124	Others n=216
**<u>Baseline Characteristics at Diagnosis</u>**			
**Demographics**			
Age, yr, median (IQR)	78 (69–85)	76 (68–83)	77 (68–84)
Female, n (%)	83 (40)	59 (48)	96 (44)
Body mass index, kg/m², median (IQR)	19.1 (17.3–22.4)	19.6 (17.3–22.4)	19.7 (16.9–23.0)
**Vital Signs and Support**			
Systolic BP, mm Hg, median (IQR)	127 (108–144)	138 (117–159)	131 (116–152)
Diastolic BP, mm Hg, median (IQR)	71 (60–85)	78 (66–93)	74 (65–85)
Vasopressor use, n (%)	11 (5)	7 (6)	3 (1)
Oxygen administration, n (%)	38 (18)	23 (19)	27 (13)
Mechanical ventilation, n (%)	2 (1)	1 (0.8)	2 (0.9)
**Pre-admission Status and Chronic Comorbidities**			
Community-onset hyponatremia, n (%)	122 (59)	87 (70)	107 (50)
Acute hyponatremia	1 (0.5)	0 (0)	1 (0.5)
Charlson comorbidity index, median (IQR)	2 (1–5)	2 (0–3)	2 (1–3)
Chronic heart failure, n (%)	52 (25)	24 (19)	33 (15)
NYHA I-II, n (%)	5 (2)	1 (0.8)	7 (3)
NYHA III-IV, n (%)	8 (4)	2 (2)	4 (2)
NYHA unknown, n (%)	39 (19)	21 (17)	22 (10)
Chronic kidney disease, n (%)	49 (24)	23 (19)	39 (18)
Maintenance dialysis, n (%)	1 (0.5)	1 (0.8)	4 (2)
Liver cirrhosis, n (%)	18 (9)	2 (2)	6 (3)
Child–Pugh A, n (%)	3 (1)	1 (0.8)	2 (0.9)
Child–Pugh B, n (%)	5 (2)	1 (0.8)	0 (0.0)
Child–Pugh C, n (%)	10 (5)	0 (0)	4 (2)
Solid tumor without metastasis, n (%)	24 (12)	12 (10)	24 (11)
Metastatic malignant tumor, n (%)	49 (24)	11 (9)	33 (15)
**Pre-admission Medications**			
Loop diuretics, n (%)	45 (22)	16 (13)	33 (15)
Thiazide diuretics, n (%)	23 (11)	20 (16)	16 (7)
SSRI/SNRI, n (%)	2 (1)	7 (6)	8 (4)
NSAIDs, n (%)	25 (12)	16 (13)	28 (13)
Opioids, n (%)	13 (6)	1 (0.8)	10 (5)

	Normal electrolyte solution only n=206	Low electrolyte solution only n=124	Others n=216
**Acute Comorbidities in 30 Days, n (%)**			
Pneumonia	41 (20)	14 (11)	39 (18)
Urinary tract infection	13 (6)	10 (8)	16 (7)
Skin and soft-tissue infection	4 (2)	3 (2)	3 (1)
Acute heart failure	15 (7)	11 (9)	20 (9)
Acute myocardial infarction	2 (1)	0 (0)	0 (0)
Stroke	6 (3)	5 (4)	5 (2)
Acute kidney injury	47 (23)	21 (17)	22 (10)
Fracture	9 (4)	7 (6)	20 (9)
**Etiology of hyponatremia, n (%)**			
Primary polydipsia	8 (4)	14 (11)	12 (6)
Hypovolemia	30 (15)	19 (15)	19 (9)
SIAD	129 (63)	71 (57)	142 (66)
Drug-related	26 (13)	29 (23)	26 (12)
Adrenal insufficiency	3 (1)	3 (2)	7 (3)
Unidentified cause	28 (14)	14 (11)	26 (12)
**Laboratory Data, median (IQR)**			
Serum Na, mEq/L (baseline)	118 (116–119)	117 (114–119)	118 (115–119)
Serum K, mEq/L	4.5 (4.0–5.2)	4.1 (3.6–4.7)	4.3 (3.9–4.7)
Albumin, g/dL	3.0 (2.5–3.6)	3.6 (2.9–4.0)	3.3 (2.7–3.9)
eGFR, mL/min/1.73 m^2	69 (39–94)	82 (49–112)	89 (60–122)
CRP, mg/dL	2.2 (0.6–8.5)	0.7 (0.1–5.9)	1.3 (0.2–4.6)
Glucose, mg/dL	122 (100–152)	129 (115–158)	122 (103–150)
White blood cell count, $\times 10^2/\mu L$	90 (60–115)	81 (57–107)	73 (55–102)
Hemoglobin, g/dL	10.8 (9.7–12.1)	11.5 (10.3–12.9)	11.2 (9.8–12.5)
Platelet count, $\times 10^4/\mu L$	21.2 (15.5–28.2)	20.5 (15.9–28.1)	21.7 (15.9–27.1)
Urinary Na, mEq/L	53 (31–83)	58 (25–83)	65 (31–99)
Urinary K, mEq/L	28 (18–39)	26 (17–42)	28 (19–44)

Abbreviations: BP, Blood Pressure; CRP, C-reactive protein; eGFR, estimated glomerular filtration rate; IQR, interquartile range; K, potassium; Na, sodium; NSAIDs, nonsteroidal anti-inflammatory drugs; NYHA, New York Heart Association; SIAD, syndrome of inappropriate antidiuresis; SSRI/SNRI, selective serotonin reuptake inhibitor/serotonin-norepinephrine reuptake inhibitor; yr, year

Supplementary Table S7. Clinical course among in-hospital deaths included in the analytic cohort

Age	Sex	Baseline Serum Na (mEq/L)	Time to Death from Diagnosis (days)	Last Measured Serum Na (mEq/L)	Cause of Death (Category)	Cause of Death (Specific)	Comfort-focused Care	Active attempt to correct serum Na	Correction Method	Cerebral edema documented	Brain herniation documented
78	Female	112	3	109	Other or Unspecified Causes	Interstitial pneumonia	No	No	NE fluid	Not evaluated	Not evaluated
80	Male	114	120	148	Infectious Diseases	Aspiration pneumonia	No	Yes	NE fluid	No	No
89	Female	116	5	151	Other or Unspecified Causes	Bilateral cerebral infarction	Yes	No	None	Not evaluated	Not evaluated
70	Male	117	9	119	Malignant Tumors	Lung cancer	Yes	No	NE fluid	Not evaluated	Not evaluated
64	Female	118	30	130	Malignant Tumors	Pancreatic cancer	Yes	Yes	NE fluid; LD	Not evaluated	Not evaluated
75	Female	118	43	137	Malignant Tumors	Bladder cancer	Yes	No	NE fluid	Not evaluated	Not evaluated
63	Male	120	16	131	Gastrointestinal Diseases	Liver cirrhosis	No	Yes	NE fluid	Not evaluated	Not evaluated
56	Female	120	16	132	Gastrointestinal Diseases	Liver cirrhosis	No	No	NE fluid	Not evaluated	Not evaluated
71	Male	120	69	132	Infectious Diseases	Pneumonia	Yes	No	NE fluid	Not evaluated	Not evaluated
83	Male	113	31	132	Infectious Diseases	Aspiration pneumonia	No	No	NE fluid	Not evaluated	Not evaluated
63	Male	116	22	123	Malignant Tumors	Lung cancer	No	Yes	NE fluid	Not evaluated	Not evaluated
66	Male	117	15	148	Malignant Tumors	Esophageal cancer	Yes	Yes	NE fluid	Not evaluated	Not evaluated
89	Female	119	30	144	Circulatory Diseases	Heart failure	Yes	Yes	LD	Not evaluated	Not evaluated
73	Male	119	90	133	Malignant Tumors	Multiple myeloma	Yes	No	NE fluid	Not evaluated	Not evaluated
67	Male	120	22	142	Infectious Diseases	Pneumonia	No	Yes	NE fluid; LD	Not evaluated	Not evaluated
80	Male	115	4	116	Malignant Tumors	Bile duct cancer	Yes	No	LE fluid	Not evaluated	Not evaluated
80	Male	114	27	127	Infectious Diseases	Pyothorax	No	Yes	NE fluid	Not evaluated	Not evaluated
87	Male	117	27	123	Malignant Tumors	Lung cancer	Yes	No	NE fluid	Not evaluated	Not evaluated
80	Female	118	41	137	Malignant Tumors	Lung cancer	Yes	Yes	NE fluid	Not evaluated	Not evaluated
97	Male	118	6	140	Gastrointestinal Diseases	Bowel obstruction	Yes	Yes	NE fluid	Not evaluated	Not evaluated

85	Male	120	40	133	Malignant Tumors	Liver cancer	No	Yes	NE fluid; LD	Not evaluated	Not evaluated
83	Male	120	52	141	Infectious Diseases	Pneumonia	No	No	None	Not evaluated	Not evaluated
63	Female	120	48	156	Malignant Tumors	Ascending colon cancer	Yes	Yes	NE fluid	Not evaluated	Not evaluated
78	Male	120	41	139	Malignant Tumors	Prostate cancer	Yes	No	NE fluid	Not evaluated	Not evaluated
70	Male	115	18	142	Malignant Tumors	Lung cancer	No	Yes	HE-cont	Not evaluated	Not evaluated
78	Male	115	12	122	Malignant Tumors	Pharyngeal cancer	Yes	No	LE fluid	Not evaluated	Not evaluated
68	Male	115	43	138	Malignant Tumors	Gastric cancer	No	Yes	HE-cont; NE fluid	Not evaluated	Not evaluated
89	Female	120	24	132	Infectious Diseases	Pulmonary tuberculosis	Yes	No	NE fluid	Not evaluated	Not evaluated
81	Male	120	25	165	Malignant Tumors	Gastric cancer	Yes	Yes	NE fluid	Not evaluated	Not evaluated
71	Male	112	9	137	Infectious Diseases	Aspiration pneumonia	No	Yes	HE-bolus; NE fluid; LE fluid	No	No
65	Female	118	13	137	Infectious Diseases	Sepsis	No	Yes	LE fluid	Not evaluated	Not evaluated
88	Male	116	12	152	Infectious Diseases	Pneumonia	No	No	NE fluid	Not evaluated	Not evaluated
47	Female	119	56	131	Circulatory Diseases	Ruptured aortic aneurysm	No	Yes	HE-cont	Not evaluated	Not evaluated
76	Male	120	51	144	Other or Unspecified Causes	Unknown cause	No	Yes	HE-cont; LE fluid; DDAVP	No	No
68	Male	118	11	157	Circulatory Diseases	Heart failure	No	Yes	NE fluid; LD; Vaptan	No	No
73	Male	119	55	129	Circulatory Diseases	Heart failure	No	Yes	NE fluid; LD; Vaptan	Not evaluated	Not evaluated
74	Male	119	5	129	Malignant Tumors	Lung cancer	Yes	No	LE fluid	Not evaluated	Not evaluated
69	Male	120	8	127	Malignant Tumors	Lung cancer	Yes	No	NE fluid	Not evaluated	Not evaluated
84	Female	120	12	131	Infectious Diseases	Pneumonia	Yes	Yes	NE fluid; LD	Not evaluated	Not evaluated
64	Female	106	8	134	Other or Unspecified Causes	CO2 narcosis	No	Yes	HE-cont; NE fluid; LE fluid; DDAVP	Not evaluated	Not evaluated
91	Female	113	86	153	Infectious Diseases	Aspiration pneumonia	Yes	No	NE fluid	Not evaluated	Not evaluated
83	Male	114	5	141	Circulatory Diseases	Heart failure	No	No	NE fluid; LD	Not evaluated	Not evaluated
78	Male	115	75	142	Other or Unspecified Causes	Unknown cause	Yes	Yes	HE-cont	Not evaluated	Not evaluated
72	Male	119	18	139	Gastrointestinal Diseases	Sigmoid colon perforation	Yes	Yes	HE-cont; NE fluid	Not evaluated	Not evaluated

93	Male	119	19	117	Circulatory Diseases	Heart failure	No	Yes	NE fluid	Not evaluated	Not evaluated
87	Female	117	48	140	Other or Unspecified Causes	Unknown cause	No	Yes	HE-cont; LE fluid	Not evaluated	Not evaluated
47	Male	119	7	131	Circulatory Diseases	Ruptured aortic aneurysm	No	No	NE fluid	Not evaluated	Not evaluated
83	Male	119	16	134	Malignant Tumors	Lung cancer	Yes	No	None	Not evaluated	Not evaluated
78	Female	120	7	124	Malignant Tumors	Liver cancer	Yes	Yes	HE-cont	Not evaluated	Not evaluated
83	Female	110	3	130	Gastrointestinal Diseases	Mesenteric ischemia	No	Yes	NE fluid; LE fluid	Not evaluated	Not evaluated
59	Female	118	43	133	Malignant Tumors	Gastric cancer	No	No	NE fluid	Not evaluated	Not evaluated
71	Male	120	8	121	Circulatory Diseases	Heart failure	No	Yes	LE fluid; LD; Vaptan	Not evaluated	Not evaluated
73	Male	120	42	176	Malignant Tumors	Pancreatic cancer	No	No	NE fluid	Not evaluated	Not evaluated
66	Male	119	66	135	Malignant Tumors	Rectal cancer	Yes	Yes	NE fluid	Not evaluated	Not evaluated
85	Male	118	16	130	Infectious Diseases	Pneumonia	Yes	No	NE fluid	Not evaluated	Not evaluated
76	Female	118	40	125	Malignant Tumors	Cervical cancer	No	Yes	HE-cont	Not evaluated	Not evaluated
91	Male	118	11	155	Circulatory Diseases	Heart failure	No	Yes	NE fluid	No	No
56	Male	110	11	137	Gastrointestinal Diseases	Liver cirrhosis	Yes	Yes	HE-bolus; HE-cont; NE fluid	No	No
72	Male	114	97	140	Infectious Diseases	Aspiration pneumonia	No	Yes	LE fluid; DDAVP	Not evaluated	Not evaluated
73	Female	116	8	128	Other or Unspecified Causes	CO2 narcosis	No	Yes	HE-bolus; HE-cont; LE fluid	Not evaluated	Not evaluated
81	Female	120	15	138	Infectious Diseases	Aspiration pneumonia	Yes	No	None	Not evaluated	Not evaluated
87	Male	120	13	145	Malignant Tumors	Ascending colon cancer	Yes	No	NE fluid	Not evaluated	Not evaluated
70	Female	120	14	121	Circulatory Diseases	Heart failure	Yes	No	LE fluid; LD	Not evaluated	Not evaluated
74	Male	114	19	139	Malignant Tumors	Lung cancer	No	Yes	HE-cont	Not evaluated	Not evaluated
91	Male	120	11	142	Malignant Tumors	Lung cancer	Yes	No	NE fluid	Not evaluated	Not evaluated
79	Female	118	58	127	Gastrointestinal Diseases	Bowel obstruction	No	No	LE fluid	Not evaluated	Not evaluated
89	Female	111	19	159	Infectious Diseases	Pyelonephritis	No	Yes	HE-cont; NE fluid; LD; Vaptan	Not evaluated	Not evaluated
77	Male	118	7	120	Gastrointestinal Diseases	Liver cirrhosis	No	No	LD	Not evaluated	Not evaluated

96	Male	117	31	141	Infectious Diseases	Aspiration pneumonia	Yes	No	None	No	No
69	Male	117	54	138	Malignant Tumors	Lung cancer	No	No	NE fluid	Not evaluated	Not evaluated
85	Female	119	20	141	Circulatory Diseases	Heart failure	No	Yes	HE-cont; NE fluid	Not evaluated	Not evaluated
70	Male	119	3	132	Other or Unspecified Causes	Interstitial pneumonia	No	Yes	HE-bolus; HE-cont	Not evaluated	Not evaluated
79	Male	119	83	126	Malignant Tumors	Renal pelvic cancer	No	Yes	HE-cont; NE fluid	Not evaluated	Not evaluated
88	Female	120	15	135	Infectious Diseases	Cholangitis	Yes	Yes	HE-cont; NE fluid	Not evaluated	Not evaluated
82	Female	119	4	123	Other or Unspecified Causes	Interstitial pneumonia	No	Yes	NE fluid	Not evaluated	Not evaluated
75	Female	119	9	122	Malignant Tumors	Bile duct cancer	Yes	Yes	Vaptan	Not evaluated	Not evaluated
88	Female	119	6	133	Infectious Diseases	Aspiration pneumonia	No	Yes	HE-cont	Not evaluated	Not evaluated
71	Male	120	15	125	Malignant Tumors	Cecal cancer	Yes	Yes	HE-cont; LD	Not evaluated	Not evaluated
77	Female	108	11	133	Malignant Tumors	Pancreatic cancer	No	Yes	HE-bolus; LE fluid	Not evaluated	Not evaluated
86	Male	119	30	155	Other or Unspecified Causes	Interstitial pneumonia	No	No	NE fluid	Not evaluated	Not evaluated
71	Female	116	14	140	Malignant Tumors	Sigmoid colon cancer	Yes	No	NE fluid	Not evaluated	Not evaluated
87	Male	119	32	142	Infectious Diseases	Aspiration pneumonia	No	Yes	HE-cont	Not evaluated	Not evaluated
88	Female	119	48	132	Circulatory Diseases	Heart failure	No	Yes	NE fluid; LD; Vaptan	Not evaluated	Not evaluated
75	Male	119	29	136	Circulatory Diseases	Heart failure	Yes	No	LD	Not evaluated	Not evaluated
78	Male	120	34	135	Infectious Diseases	Aspiration pneumonia	No	Yes	NE fluid	Not evaluated	Not evaluated
73	Male	119	17	127	Malignant Tumors	Hepatocellular carcinoma	Yes	No	NE fluid; LD	Not evaluated	Not evaluated
88	Female	120	28	123	Infectious Diseases	Aspiration pneumonia	Yes	No	NE fluid	Not evaluated	Not evaluated
73	Male	120	8	130	Infectious Diseases	Pyelonephritis	Yes	No	NE fluid	No	No
81	Female	117	11	126	Malignant Tumors	Hepatocellular carcinoma	Yes	Yes	HE-cont	Not evaluated	Not evaluated
74	Male	118	21	131	Malignant Tumors	Gastric cancer	Yes	No	NE fluid	Not evaluated	Not evaluated
77	Male	119	30	139	Malignant Tumors	Hepatocellular carcinoma	Yes	No	NE fluid	Not evaluated	Not evaluated
83	Male	120	26	134	Other or Unspecified Causes	End-stage renal failure	Yes	No	NE fluid	Not evaluated	Not evaluated

69	Male	115	30	144	Malignant Tumors	Gastric cancer	No	Yes	HE-bolus; HE-cont	No	No
73	Male	119	8	132	Malignant Tumors	Pancreatic cancer	Yes	No	NE fluid	Not evaluated	Not evaluated
80	Female	118	53	136	Other or Unspecified Causes	Interstitial pneumonia	No	No	None	Not evaluated	Not evaluated
85	Female	107	8	125	Circulatory Diseases	Heart failure	No	Yes	HE-bolus; NE fluid	Not evaluated	Not evaluated
47	Male	119	3	116	Gastrointestinal Diseases	Liver cirrhosis	Yes	No	NE fluid	Not evaluated	Not evaluated
86	Female	119	13	152	Infectious Diseases	Aspiration pneumonia	No	Yes	LD	Not evaluated	Not evaluated
83	Female	118	9	130	Malignant Tumors	Pancreatic cancer	Yes	Yes	NE fluid	Not evaluated	Not evaluated

The median age was 78 years (IQR, 71–85), and the median baseline serum Na level was 119 mEq/L (IQR, 116–120). The median time from diagnosis to death was 18 days (IQR, 10–40), and the median last measured serum Na level was 134 mEq/L (IQR, 129–141). The causes of death were malignant tumors in 40 patients (40%), infectious diseases in 24 (24%), circulatory diseases in 14 (14%), gastrointestinal diseases in 9 (9%), and other or unspecified causes in 12 (12%). No deaths were attributed primarily to hyponatremia based on clinical documentation. Comfort-focused care was documented in 48 patients (48%). An active attempt to correct serum Na was documented in 55 patients (56%) and was not documented in 44 (44%). No documented cerebral edema or brain herniation was identified; for both outcomes, 9 patients (9%) were evaluated and had no documented findings, whereas 90 (91%) were not evaluated.

Comfort-focused care was defined as documented best supportive care, terminal care, palliative care, or a care plan prioritizing symptom relief without treatment escalation, recorded at or before 24 h after diagnosis. DNR/DNAR status alone was not considered comfort-focused care. Active attempts to correct serum Na were defined as treatments used with the intent of managing serum Na correction. Initiation of HE-bolus, HE-cont, vaptan, or DDAVP was considered an active correction attempt regardless of documentation. NE fluid, LE fluid, and LD were considered active correction attempts only when correction of serum Na was documented as the treatment purpose. Cerebral edema and brain herniation were considered documented only when described in CT, MRI, autopsy findings, or physician documentation.

Abbreviations: DDAVP, desmopressin; DNR/DNAR, do-not-resuscitate/do-not-attempt-resuscitation; HE-bolus, bolus high-electrolyte solution; HE-cont, continuous high-electrolyte solution; IQR, interquartile range; LD, loop diuretic; LE fluid, low-electrolyte solution; Na, sodium; NE fluid, normal-electrolyte solution.

Supplementary Table S8. Clinical course among patients who died within 24 hours after hyponatremia diagnosis

Age	Sex	Baseline Serum Na (mEq/L)	Time to Death from Diagnosis (hours)	Last Measured Serum Na (mEq/L)	Cause of Death (Category)	Cause of Death (Specific)	Comfort-focused Care	Active attempt to correct serum Na	Correction Method	Cerebral edema documented	Brain herniation documented
72	Male	116	22	Not repeated	Malignant Tumors	Lung cancer	Yes	No	LE fluid	Not evaluated	Not evaluated
37	Female	120	4	Not repeated	Gastrointestinal Diseases	Liver cirrhosis	Yes	No	NE fluid	No	No
77	Male	120	7	Not repeated	Infectious Diseases	Pneumonia	No	No	LE fluid	Not evaluated	Not evaluated
60	Male	117	1	Not repeated	Malignant Tumors	Bile duct cancer	Yes	No	LE fluid	Not evaluated	Not evaluated
69	Male	118	20	Not repeated	Malignant Tumors	Hepatocellular carcinoma	Yes	No	NE fluid	Not evaluated	Not evaluated
65	Female	120	4	Not repeated	Malignant Tumors	Breast cancer	Yes	No	NE fluid	No	No
50	Female	119	8	Not repeated	Malignant Tumors	Ovarian cancer	Yes	No	NE fluid	Not evaluated	Not evaluated
80	Male	116	11	Not repeated	Malignant Tumors	Lung cancer	Yes	No	LE fluid	Not evaluated	Not evaluated
35	Female	119	19	127	Circulatory Diseases	Heart failure	No	Yes	HE-cont; LD	Not evaluated	Not evaluated
78	Female	116	2	Not repeated	Malignant Tumors	Pancreatic cancer	Yes	No	LE fluid	Not evaluated	Not evaluated
92	Male	118	8	Not repeated	Infectious Diseases	Sepsis	No	No	NE fluid	Not evaluated	Not evaluated
36	Female	120	4	Not repeated	Other or Unspecified Causes	Intrathoracic hemorrhage	Yes	No	NE fluid	No	No
88	Female	116	23	118	Circulatory Diseases	Heart failure	Yes	Yes	NE fluid; LD	No	No
65	Male	119	6	Not repeated	Gastrointestinal Diseases	Liver cirrhosis	Yes	No	NE fluid	Not evaluated	Not evaluated
48	Male	118	23	Not repeated	Malignant Tumors	Lung cancer	Yes	No	LE fluid	Not evaluated	Not evaluated
92	Female	106	23	Not repeated	Circulatory Diseases	Heart failure	Yes	No	NE fluid; LD	No	No
37	Female	120	15	Not repeated	Malignant Tumors	Descending colon cancer	Yes	No	NE fluid	Not evaluated	Not evaluated
88	Male	119	6	Not repeated	Gastrointestinal Diseases	Liver cirrhosis	Yes	No	NE fluid	No	No
59	Male	113	14	Not repeated	Infectious Diseases	Pneumonia	Yes	No	NE fluid	Not evaluated	Not evaluated
85	Female	117	23	Not repeated	Circulatory Diseases	Heart failure	Yes	No	LE fluid	No	No

The median age was 67 years (IQR, 50–81), and the median baseline serum Na level was 118 mEq/L (IQR, 116–119). The median time from diagnosis to death was 10 h (IQR, 6–21). Serum Na was not repeated before death in 18 patients (90%); the last measured serum Na levels in the remaining two patients were 127 and 118 mEq/L. The causes of death were malignant tumors in 9 patients (45%), circulatory diseases in 4 (20%), infectious diseases in 3 (15%), gastrointestinal diseases in 3 (15%), and other cause in 1 (5%). No deaths were attributed primarily to hyponatremia based on clinical documentation. Comfort-focused care was documented in 17 patients (85%). An active attempt to correct serum Na was documented in 2 patients (10%) and was not documented in 18 (90%). No documented cerebral edema or brain herniation was identified; for both outcomes, 7 patients (35%) were evaluated and had no documented findings, whereas 13 (65%) were not evaluated.

Comfort-focused care was defined as documented best supportive care, terminal care, palliative care, or a care plan prioritizing symptom relief without treatment escalation, recorded at or before death. DNR/DNAR status alone was not considered comfort-focused care. Active attempts to correct serum Na were defined as treatments used with the intent of managing serum Na correction. Initiation of HE-bolus, HE-cont, vaptan, or DDAVP was considered an active correction attempt regardless of documentation. NE fluid, LE fluid, and LD were considered active correction attempts only when correction of serum Na was documented as the treatment purpose. Cerebral edema and brain herniation were considered documented only when described in CT, MRI, autopsy findings, or physician documentation.

Abbreviations: DDAVP, desmopressin; DNR/DNAR, do-not-resuscitate/do-not-attempt-resuscitation; HE-bolus, bolus high-electrolyte solution; HE-cont, continuous high-electrolyte solution; IQR, interquartile range; LD, loop diuretic; LE fluid, low-electrolyte solution; Na, sodium; NE fluid, normal-electrolyte solution.

Supplementary Figure S1. Patient selection flowchart

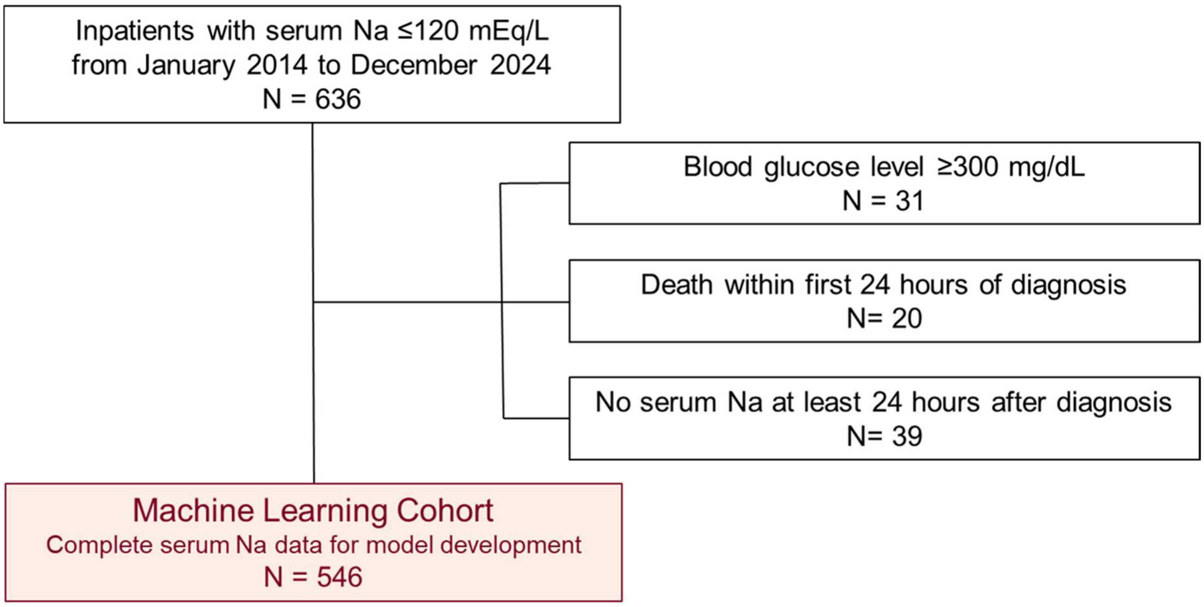

Supplementary Figure S2. Distribution of 24-hour changes in the serum sodium level

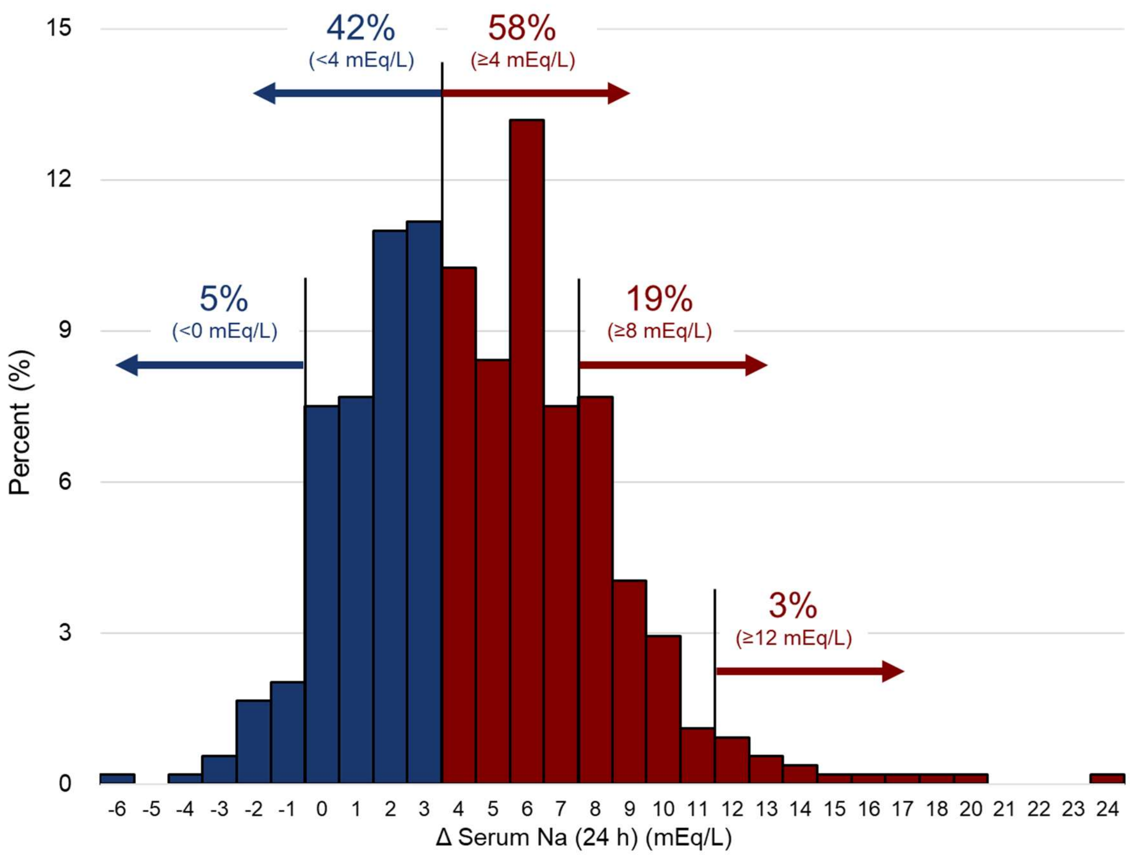

Supplementary Figure S3. Spearman correlation matrix (variables with |r|>0.5)

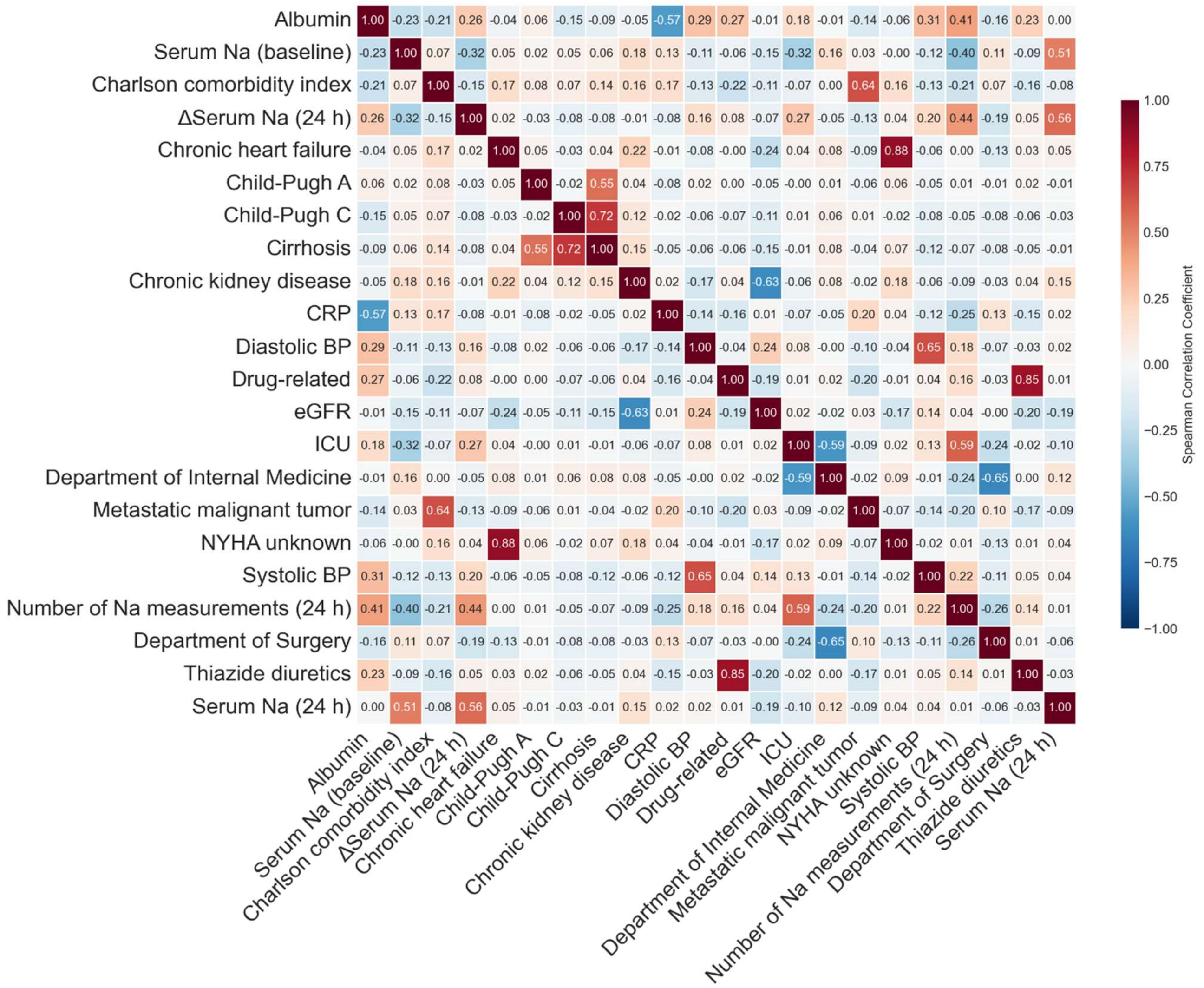

Supplementary Figure S4. Features selected by each machine learning classifier

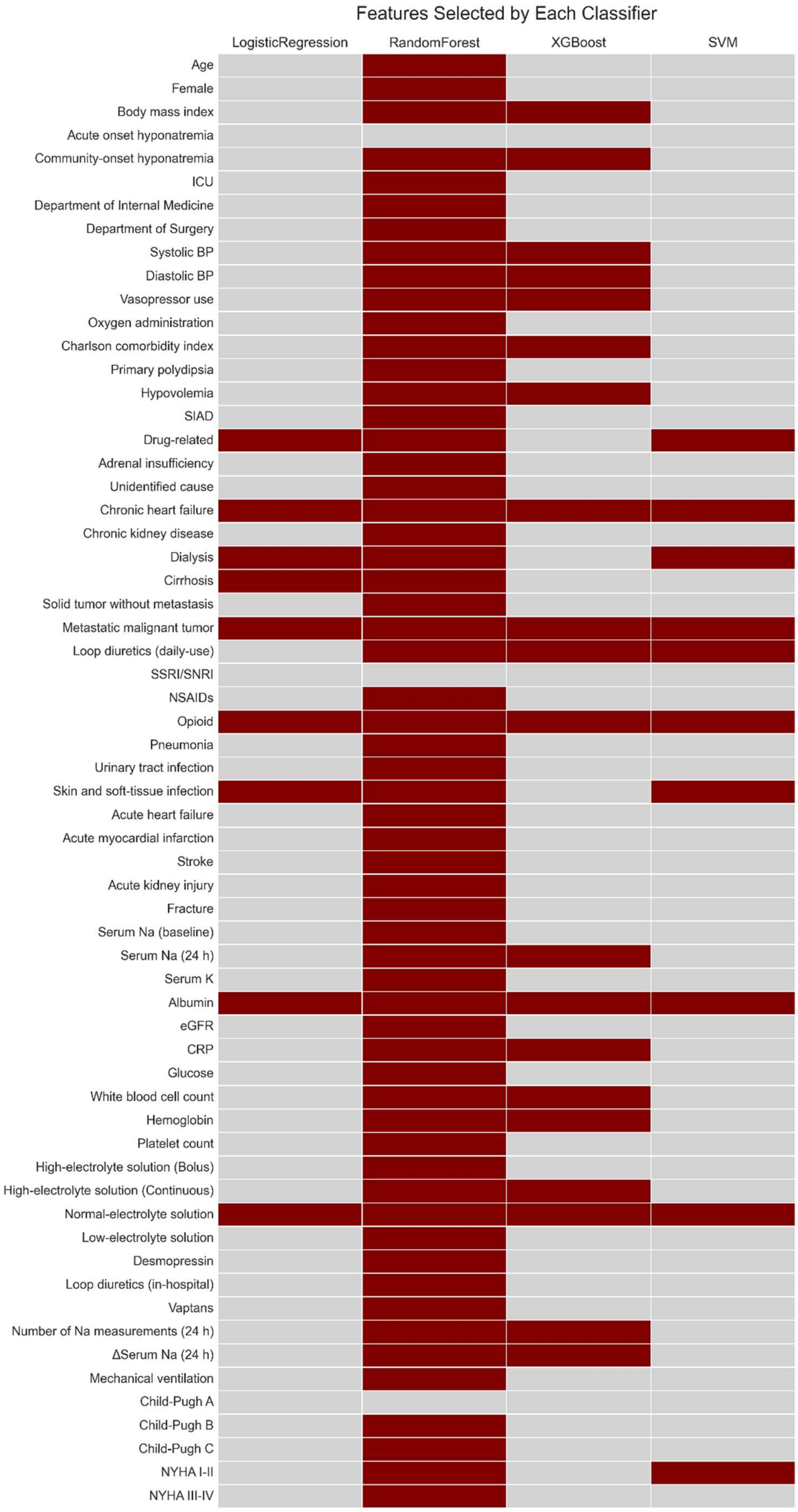

Supplementary Figure S5. SHAP dependence plots for all selected features (full model)

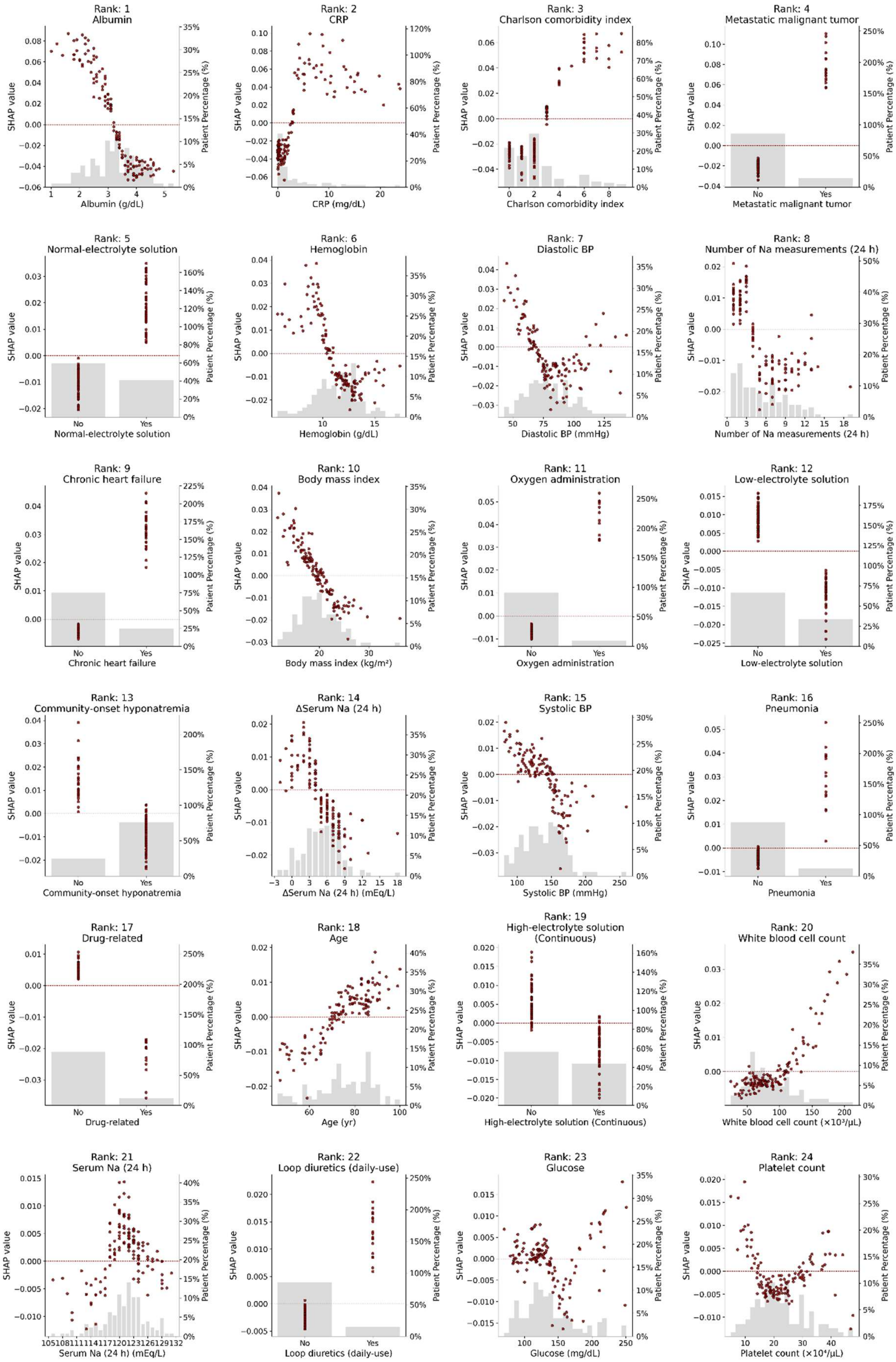

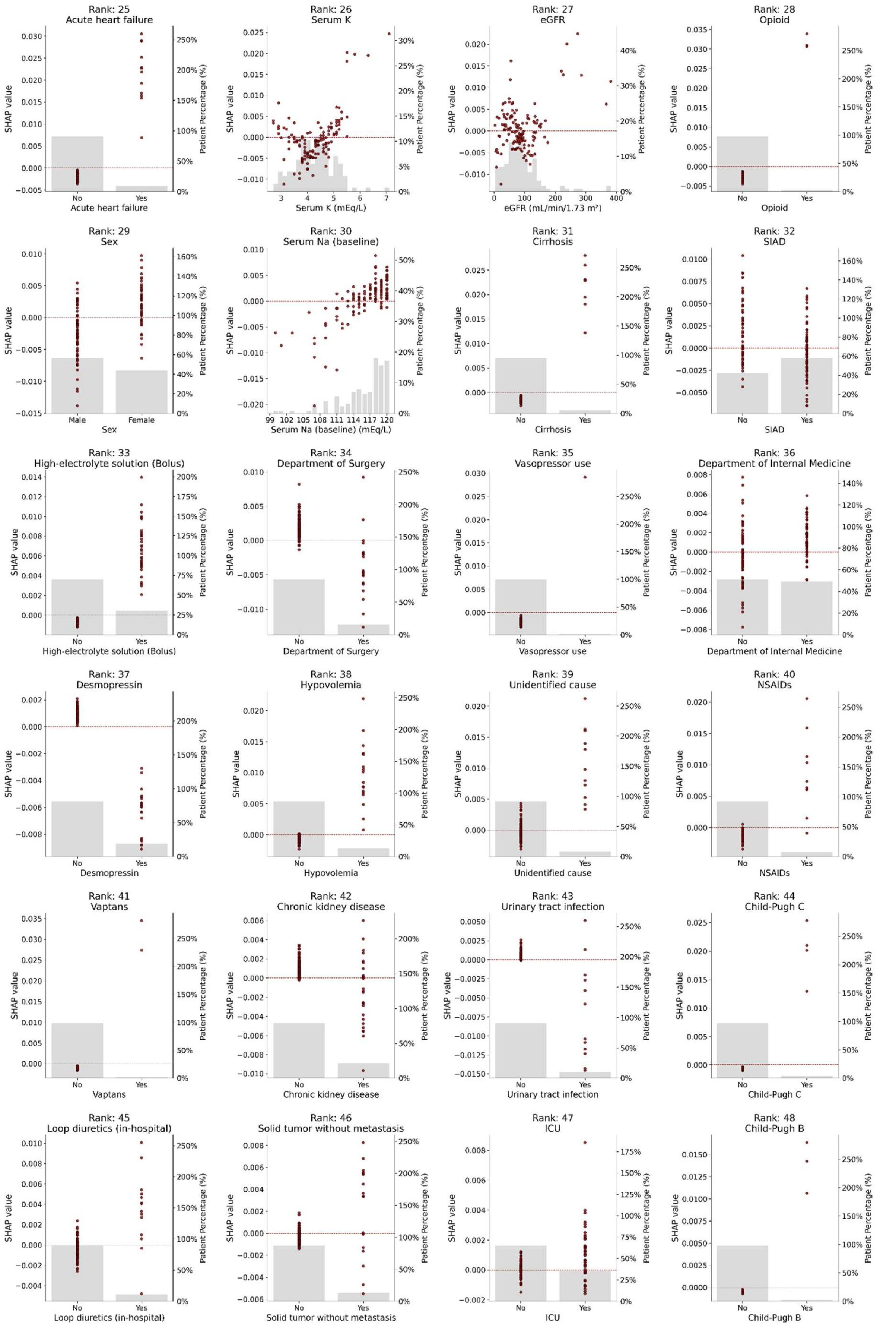
Rank: 25
Acute heart failure
Rank: 26
Serum K
Serum K (mEq/L)
Rank: 27
eGFR
eGFR (mL/min/1.73 m²)
Rank: 28
Opioid
Rank: 29
Sex
Male
Female
Rank: 30
Serum Na (baseline)
Serum Na (baseline) (mEq/L)
Rank: 31
Cirrhosis
Rank: 32
SIAD
Rank: 33
High-electrolyte solution (Bolus)
Rank: 34
Department of Surgery
Rank: 35
Vasopressor use
Rank: 36
Department of Internal Medicine
Rank: 37
Desmopressin
Rank: 38
Hypovolemia
Rank: 39
Unidentified cause
Rank: 40
NSAIDs
Rank: 41
Vaptans
Rank: 42
Chronic kidney disease
Rank: 43
Urinary tract infection
Rank: 44
Child-Pugh C
Rank: 45
Loop diuretics (in-hospital)
Rank: 46
Solid tumor without metastasis
Rank: 47
ICU
Rank: 48
Child-Pugh B
SHAP value
Patient Percentage (%)
No
Yes

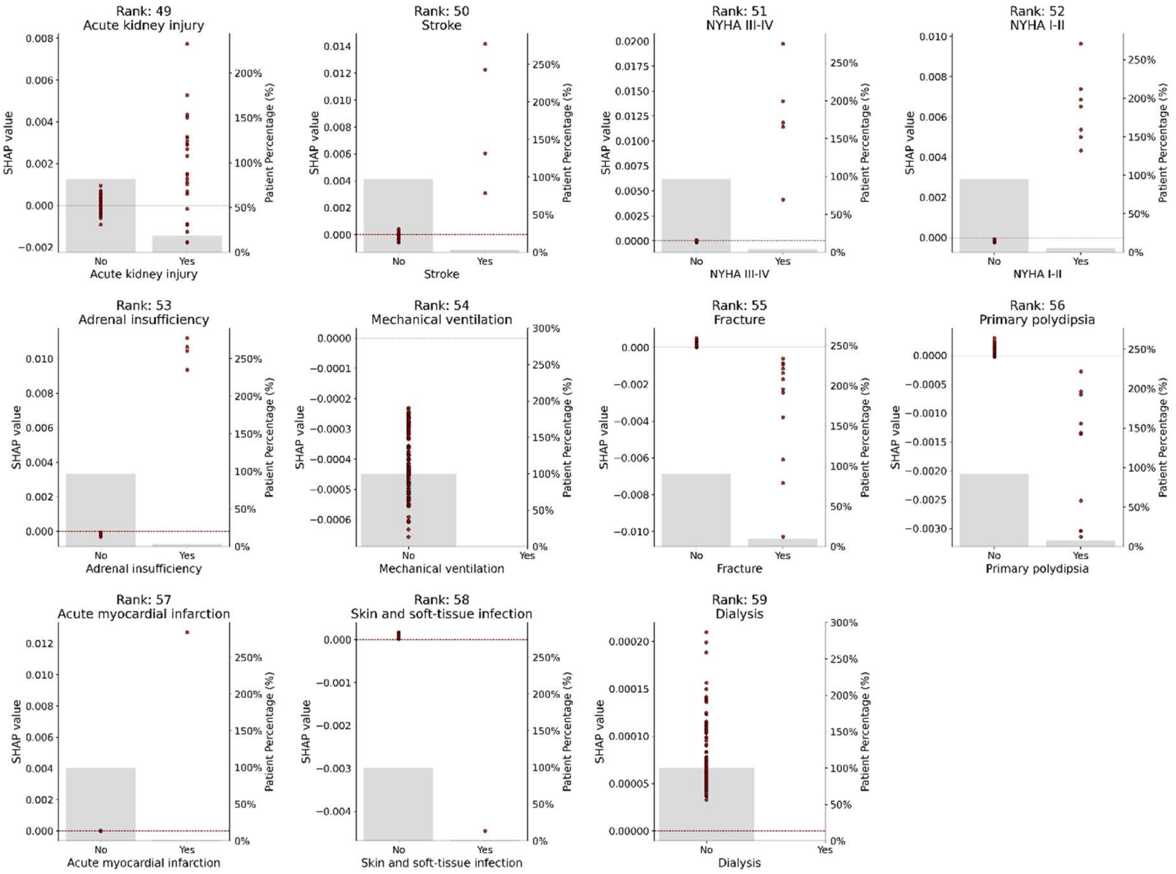
Rank: 49
Acute kidney injury
Rank: 50
Stroke
Rank: 51
NYHA III-IV
Rank: 52
NYHA I-II
Rank: 53
Adrenal insufficiency
Rank: 54
Mechanical ventilation
Rank: 55
Fracture
Rank: 56
Primary polydipsia
Rank: 57
Acute myocardial infarction
Rank: 58
Skin and soft-tissue infection
Rank: 59
Dialysis
SHAP value
Patient Percentage (%)
No
Yes

Supplementary Figure S6. SHAP dependence plots for all selected features (baseline model)

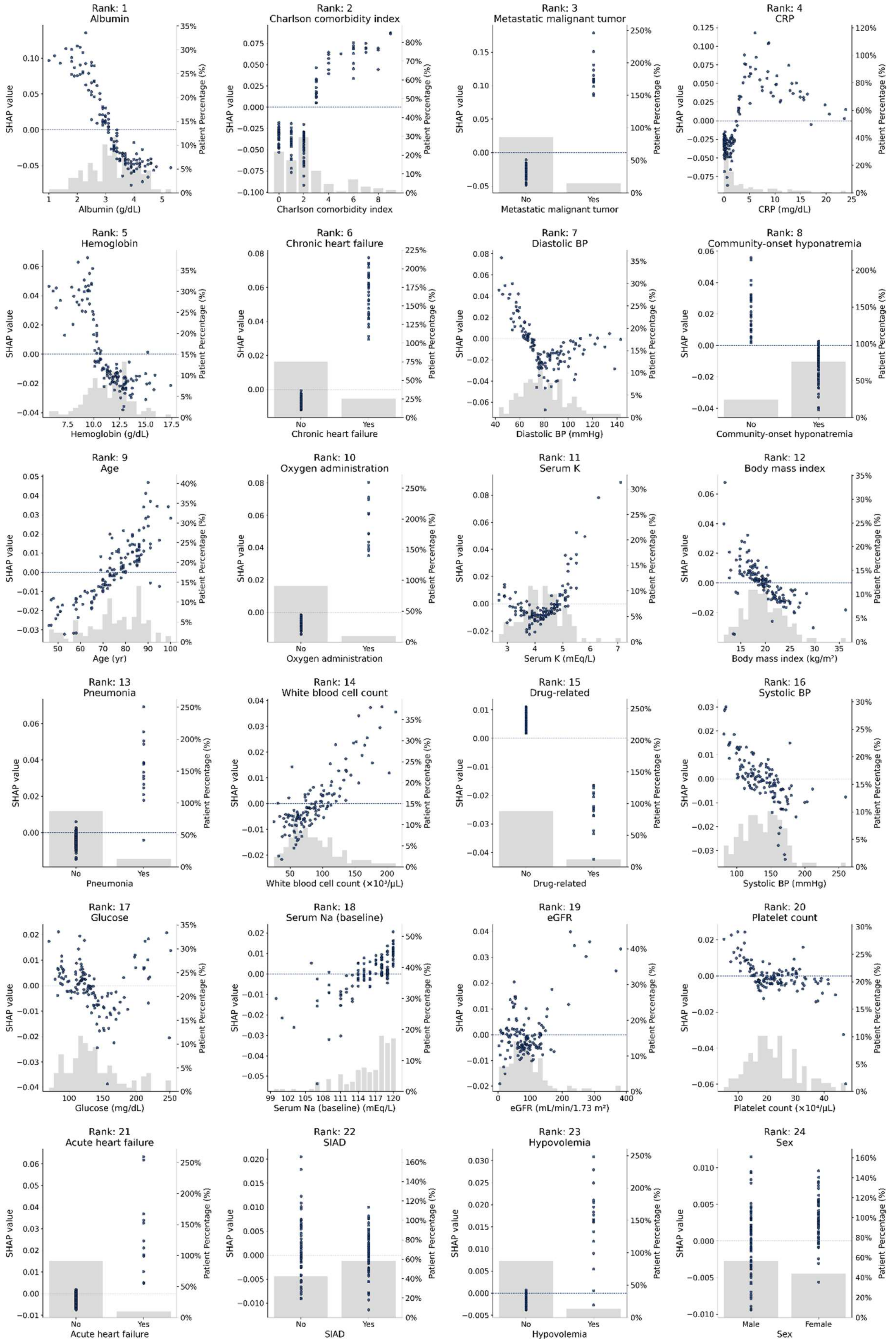

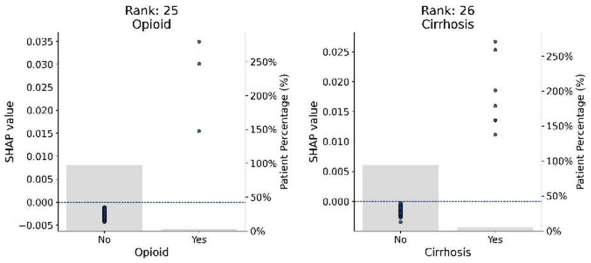
Rank: 25
Opioid
SHAP value
Patient Percentage (%)
No
Yes
Opioid
Rank: 26
Cirrhosis
SHAP value
Patient Percentage (%)
No
Yes
Cirrhosis
